# Multiple spillovers from humans and onward transmission of SARS-CoV-2 in white-tailed deer

**DOI:** 10.1073/pnas.2121644119

**Published:** 2022-01-25

**Authors:** Suresh V. Kuchipudi, Meera Surendran-Nair, Rachel M. Ruden, Michele Yon, Ruth H. Nissly, Kurt J. Vandegrift, Rahul K. Nelli, Lingling Li, Bhushan M. Jayarao, Costas D. Maranas, Nicole Levine, Katriina Willgert, Andrew J. K. Conlan, Randall J. Olsen, James J. Davis, James M. Musser, Peter J. Hudson, Vivek Kapur

**Affiliations:** ^a^Animal Diagnostic Laboratory, Department of Veterinary and Biomedical Sciences, The Pennsylvania State University, University Park, PA 16802;; ^b^Huck Institutes of Life Sciences, The Pennsylvania State University, University Park, PA 16802;; ^c^Wildlife Bureau, Iowa Department of Natural Resources, Des Moines, IA 50319;; ^d^Department of Veterinary Diagnostic and Production Animal Medicine, College of Veterinary Medicine, Iowa State University, Ames, IA 50011;; ^e^The Center for Infectious Disease Dynamics, Department of Biology and Huck Institutes of the Life Sciences, The Pennsylvania State University, University Park, PA 16802;; ^f^Department of Chemical Engineering, The Pennsylvania State University, University Park, PA 16802;; ^g^Department of Animal Science, The Pennsylvania State University, University Park, PA 16802;; ^h^Disease Dynamics Unit, Department of Veterinary Medicine, University of Cambridge, Cambridge CB3 0ES, United Kingdom;; ^i^Laboratory of Molecular and Translational Human Infectious Disease Research, Center for Infectious Diseases, Department of Pathology and Genomic Medicine, Houston Methodist Research Institute, Houston Methodist Hospital, Houston, TX 77030;; ^j^Department of Pathology and Laboratory Medicine, Weill Cornell Medical College, New York, NY 10021;; ^k^Department of Microbiology and Immunology, Weill Cornell Medical College, New York, NY 10021;; ^l^University of Chicago Consortium for Advanced Science and Engineering, University of Chicago and Division of Data Science and Learning, Argonne National Laboratory, Lemont, IL 60439

**Keywords:** SARS-CoV-2, deer, spillover, One Health, animal reservoir

## Abstract

Many animal species are susceptible to severe acute respiratory syndrome coronavirus 2 (SARS-CoV-2) infection and could act as reservoirs; however, transmission in free-living animals has not been documented. White-tailed deer, the predominant cervid in North America, are susceptible to SARS-CoV-2 infection, and experimentally infected fawns can transmit the virus. To test the hypothesis that SARS-CoV-2 is circulating in deer, 283 retropharyngeal lymph node (RPLN) samples collected from 151 free-living and 132 captive deer in Iowa from April 2020 through January of 2021 were assayed for the presence of SARS-CoV-2 RNA. Ninety-four of the 283 (33.2%) deer samples were positive for SARS-CoV-2 RNA as assessed by RT-PCR. Notably, following the November 2020 peak of human cases in Iowa, and coinciding with the onset of winter and the peak deer hunting season, SARS-CoV-2 RNA was detected in 80 of 97 (82.5%) RPLN samples collected over a 7-wk period. Whole genome sequencing of all 94 positive RPLN samples identified 12 SARS-CoV-2 lineages, with B.1.2 (*n* = 51; 54.5%) and B.1.311 (*n* = 19; 20%) accounting for ∼75% of all samples. The geographic distribution and nesting of clusters of deer and human lineages strongly suggest multiple human-to-deer transmission events followed by subsequent deer-to-deer spread. These discoveries have important implications for the long-term persistence of the SARS-CoV-2 pandemic. Our findings highlight an urgent need for a robust and proactive “One Health” approach to obtain enhanced understanding of the ecology, molecular evolution, and dissemination of SARS-CoV-2.

## Significance

The results provide strong evidence of extensive SARS-CoV-2 infection of white-tailed deer, a free-living wild animal species with widespread distribution across North, Central, and South America. The analysis shows infection of deer resulted from multiple spillovers from humans, followed by efficient deer-to-deer transmission. The discovery of widespread infection of white-tailed deer indicates their establishment as potential reservoir hosts for SARS-CoV-2, a finding with important implications for the ecology, long-term persistence, and evolution of the virus, including the potential for spillback to humans.

Severe acute respiratory syndrome coronavirus 2 (SARS-CoV-2), the cause of COVID-19 in humans, is a novel coronavirus in the genus *Betacoronavirus* (subgenus *Sarbecovirus*) (1). SARS-CoV-2 was first identified in Wuhan, China, toward the end of 2019 (2), and has caused a pandemic with more than 250 million COVID-19 cases and over 5 million deaths globally as of November 2021 (3). The virus continues to evolve and spread, with a growing concern for the emergence of new variants. SARS-CoV-2 uses the host angiotensin-converting enzyme 2 (ACE-2) receptor to enter cells (4). ACE-2 receptors are well conserved across vertebrate species, including humans (5), and computational analyses predict high binding affinities of SARS-CoV-2 to the ACE-2 receptor in multiple animal species, indicating potential susceptibility to infection (5). Included among these are three species of cervids: the Père David’s deer (*Elaphurus davidianus*), reindeer (*Rangifer tarandus*), and white-tailed deer (*Odocoileus virginianus*) (6).

Global dissemination of SARS-CoV-2 among humans provides opportunities for spillover transmission into nonhuman hosts (5, 7). Consistent with this idea, SARS‐CoV‐2 infections have been documented in dogs, cats, zoo animals, and farmed mink (8, 9). In principle, SARS-CoV-2 infection of a nonhuman animal host might result in establishing a reservoir that can further drive the emergence of novel variants with the potential for spillback to humans. This type of transmission cycle has been described among workers on mink farms (10). However, widespread SARS-CoV-2 transmission in a free-living animal species has not yet been documented.

Our study was prompted by a recent report that 40% of free-living white-tailed deer in the United States had antibodies against SARS-CoV-2 (11). Recent studies have also provided evidence of SARS-CoV-2 transmission among experimentally infected deer (6, 12). To test the hypothesis that infection and subsequent transmission of SARS-CoV-2 in deer occurs in nature, we assayed 283 retropharyngeal lymph node (RPLN) samples collected from free-living and captive deer in Iowa from April 2020 through January 2021. Sequence analysis of SARS-CoV-2 genomes in all 94 positive samples discovered multiple lineages corresponding to viral genotypes circulating contemporaneously in humans. In the aggregate, our results are consistent with a model of multiple independent human-to-deer transmission events followed by subsequent deer-to-deer transmission. The findings raise the concerning possibility of reverse zoonoses, especially in exurban areas with high deer density. Our results also highlight the potential risks and considerable knowledge gaps associated with the continued molecular evolution of SARS-CoV-2 in animal hosts. Implementation of enhanced surveillance programs to define the magnitude of the deer reservoir problem and determine whether other reservoir species exist at the animal–human interface is warranted.

## Results and Discussion

### SARS-CoV-2 RNA is Present in a Substantial Fraction of White-Tailed Deer across Iowa.

**Fig. 1.**
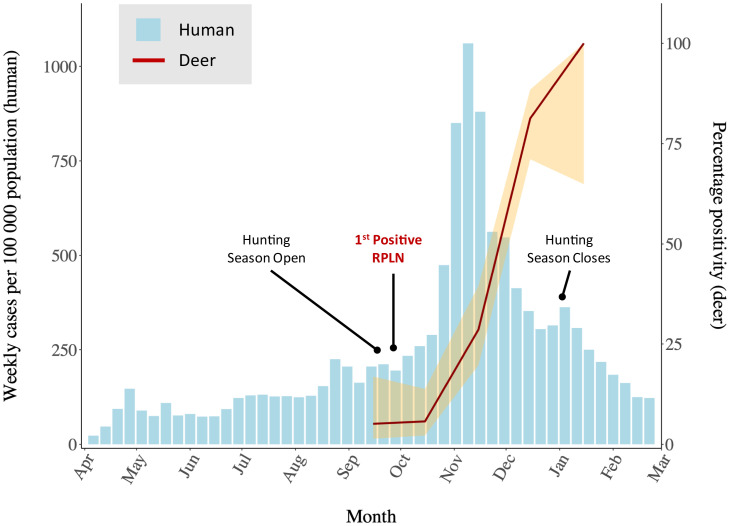
Epidemic curve showing SARS-CoV-2 weekly cases (per 100,000) in humans and the monthly change in SARS-CoV-2 positivity in white-tailed deer in Iowa. Weekly reported SARS-CoV-2 cases in humans per 100,000 population in Iowa from 30 March 2020 to 7 March 2021 (blue bars; left *y* axis) and monthly SARS-CoV-2 test positivity with 95% CI in white-tailed deer from September 2020 to January 2021 (red line; right *y* axis). The timing of the first positive sample identified in white-tailed deer on 28 September 2020 is marked, as are the start and end of the white-tailed deer hunting season on 19 September 2020 and 10 January 2021, respectively. Deer, white-tailed deer.

A total of 283 RPLN samples recovered from white-tailed deer, either free living on public lands or in periurban environments or living in managed settings (“captive”) such as hunting preserves in Iowa between 8 April 2020 and 6 January 2021, were analyzed by RT-PCR for the presence of SARS-CoV-2 RNA (13) (*SI Appendix*, Table S1). The sampling period closely follows the trajectory of the pandemic in Iowa and includes the 2020 white-tailed deer hunting season (3) (Fig. 1). Sources and characteristics of the 283 RPLN samples are summarized in Table 1. Overall, 94 of the 283 (33.2%; 95% CI: 28, 38.9) RPLN samples were found to be positive for SARS-CoV-2 RNA. A majority (*n* = 261, 91.9%) of the 283 RPLN samples in our study were harvested from deer from September through December of 2020, a period that coincided with the regular deer hunting season in Iowa that started on 19 September 2020 and ended 10 January 2021.

**Table 1.** Characteristics, demographics, and recovery of SARS-CoV-2 lineages from white-tailed deer screened for the presence of SARS-CoV-2 in Iowa

	Negative samples (N)	Positive samples (N)	Total samples (N)	Proportion/prevalence (%) (95% CI)
**Month**				
April 2020	7		7	
May 2020	4		4	
June 2020	4		4	
August 2020	2		2	
September 2020	37	2	39	5.13 (1.42–16.9)
October 2020	66	4	70	5.71 (2.24–13.8)
November 2020	55	22	77	28.6 (19.7–39.5)
December 2020	14	61	75	81.3 (71.1–88.5)
January 2021	0	5	5	100 (56.6–100)
Total	189	94	283	33.2 (28.0–38.9)
**Sex**				
Male (M)		59	179	33.0 (26.1–39.9)*
Female (F)		35	103	34.0 (24.8–43.1)*
Unknown			1	
Total		94	283	33.2 (28.0–38.9)
**Age**				
Adult (A)		82	250	32.8 (26.9–38.6)^†^
Yearling (Y)		12	32	37.5 (20.7–54.3)^†^
Fawn			1	
Total		94	283	33.2 (28.0–38.9)
**Status**				
Captive (C)		27	131	20.6 (13.7–27.5)^‡^
Free living (FL)		67	152	44.1 (36.1–51.8)^‡^
Total		94	283	33.2 (28.0–38.9)
**County**				
Allamakee		11	11	100 (74.1–100)
Jasper		4	4	100 (51.0–100)
Polk		6	7	85.7 (48.7–97.4)
Appanoose		21	28	75 (56.6–87.3)
Fayette		6	10	60 (31.3–83.2)
Jefferson		4	8	50 (21.5–78.5)
Woodbury		13	46	28.3 (17.3–42.6)
Pottawattamie		5	22	22.8 (10.1–43.4)
Des Moines		23	112	20.5 (1.5–28.9)
Dubuque		1	12	8.3 (1.5–35.4)
Black Hawk			9	0
Dickinson			1	0
Henry			6	0
Jackson			3	0
Keokuk			1	0
Van Buren			1	0
Washington			1	0
Webster			1	0
Total		94	283	33.2 (28.0–38.9)
**Lineage**				
B.1		7		7.5 (3.7–14.6)
B.1.1		1		
B.1.119		2		
B.1.2		51		54.2 (44.2–64.0)
B.1.234		6		6.4 (3.0–13.2)
B.1.240		1		
B.1.264		1		
B.1.311		19		20.2 (13.3–29.4)
B.1.362		2		
B.1.400		2		
B.1.459		1		
B.1.596		1		
Total		94		

^*^P = 0.86 (M, F).

^†^*P* = 0.59 (A, Y).

^‡^*P* < 0.0001 (C, FL).

### Strong Evidence of Temporal Clustering among the Positive Deer Samples.

**Fig. 2.**
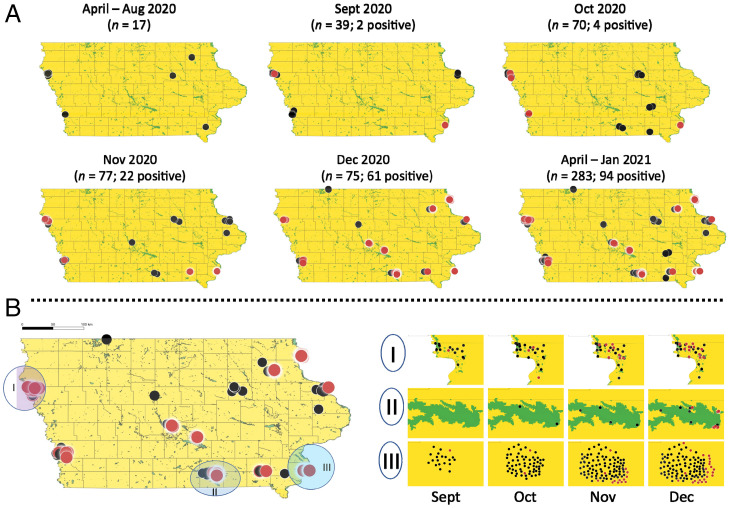
Temporal and spatial distribution of SARS-CoV-2 positive RPLN samples from white-tailed deer in Iowa. The 94 SARS-CoV-2 positive RPLN samples show strong temporal clustering in frequency of detection. (*A*) Monthly snapshots showing number and location of SARS-CoV-2 positive RPLNs from white-tailed deer. (*B*) Progression in number of positive cases in white-tailed deer from September through December 2020 in three exemplar regions in Iowa (*Left*) highlighting different sampling intensities and geographic sizes of sampling areas but similar trends of increase in incidence during November and December of 2020. Each black circle represents a negative test result, and each red circle represents a positive test result for presence of SARS-CoV-2 RNA in RPLNs. Due to geographic proximity of site of sample collection, all individual points overlaps are not visible on the state map depicted on *Left* and are highlighted for selected regions in *Right*.

We explored temporal trends in the recovery of SARS-CoV-2 RNA from deer in the sample set. The 17 RPLNs from deer collected during April through August 2020 were negative for the presence of SARS-CoV-2 RNA (Table 1). The first identification of SARS-CoV-2 in deer was on 28 September 2020, in a captive deer harvested on a game preserve in southeastern Iowa (Fig. 2 and *SI Appendix*, Table S1). This was closely followed by a second positive sample identified in a free-living deer killed in a road accident on 30 September 2020, ∼300 miles away in Woodbury County on the state’s western border (*SI Appendix*, Table S1). In total, 2 of the 39 samples collected in September were positive for SARS-CoV-2 RNA (5.1%: 95% CI: 1.4, 16.9). Similarly, in October 2020, 4 of 70 (5.7%; 95% CI 2.2, 13.8) RPLNs recovered from deer, one each from Des Moines and Pottawattamie counties, and two from Woodbury, were found to be positive for SARS-CoV-2 RNA. Coinciding with the major peak of infection in humans in Iowa, the positivity rate in deer rapidly increased, with 22 of 77 RPLN samples (27.8%; 95% CI 19.2, 38.6) harboring SARS-CoV-2 RNA in November 2020, and 61 of 75 samples (81.3%; 95% CI: 71.1, 88.5) positive in December of 2020 (Fig. 1). During the second week of January 2021, at the end of the regular hunting season, all five deer RPLN samples were positive for SARS-CoV-2 RNA (95% CI: 56.6, 100). Notably, during the 7 wk starting 23 November 2020 through the end of hunting season on 10 January 2021, 80 of 97 (82.5%; 95% CI: 73.7, 88.8) deer RPLN samples from across the state were positive for SARS-CoV-2 RNA. Importantly, the results show a high viral RNA copy number in deer RPLN samples (ranging from 2.7 copies to 2.3 × 10^6^ copies per milliliter) (*SI Appendix*, Fig. S1), suggesting that many of the deer likely had a high viral load.

### Ecological Associations and Risk Factors for Recovery of SARS-CoV-2 RNA from Free-Living and Captive Deer in Iowa.

An exploratory analysis of potential risk factors for the identification of SARS-CoV-2 RNA in deer was carried out. We found no statistically significant differences (95% level) in the proportion of positive tests by age or sex within this sample (Table 1 and *SI Appendix*, Fig. S2). However, further investigations may be warranted, since this study was not designed or powered to probe this question with rigor.

The deer included in the study were either “free living” on public lands or in periurban environments (*n* = 151) or “captive,” residing on hunting preserves (*n* = 132) (Table 1). Notably, the results suggest that the proportion of free-living deer positive for SARS-CoV-2 (44.4%; 95% CI: 36.4, 52.3) was significantly higher (z = 4.3; *P* < 0.0001) than in the deer living within preserves (20.4%; 95% CI 13.7, 27.4). However, these results may be confounded in that 4 times as many RPLN were harvested from free-living deer (*n* = 52) as were harvested from captive deer (*n* = 13) during December 2020, when the virus positivity rate was at its peak in the Iowa deer herd. Hence, further studies are needed to assess the true significance or reasons for the observed differences in prevalence between free-living and captive deer. For instance, to better assess the risk of spillover and transmission, it will be important to understand whether deer in managed settings are less stressed, have different nutritional status, or otherwise exhibit behaviors that influence opportunities for spillover or within-herd transmission of SARS-CoV-2 compared with those that are free living. Preserve deer also may have fewer exposures to people, since they are not as accessible to the broader public as are free-living deer on public lands.

We next explored regional differences in observed SARS-CoV-2 positivity among deer at the county level across Iowa. Fig. 2*A* shows the widespread distribution of positive samples recovered from deer throughout Iowa and illustrates a strong temporal trend in SARS-CoV-2 positivity as the year progressed. The study identified 10 counties with at least one positive sample (Table 1). The largest number of RPLN samples represented in the collection were from a single game preserve (preserve 2; Fig. 2*B*) in southeastern Iowa. Overall, 23 of the 112 deer RPLN samples from this preserve were found to be positive for SARS-CoV-2 RNA, with the first positive in September and the second in October 2020, and 11 of 38 deer sampled in November and all 10 deer sampled in December 2020, suggesting a rapidly increasing herd-level prevalence.

Seven counties had at least 10 samples collected, with all 11 specimens from Allamakee County being found to be SARS-CoV-2 positive, as were 21 of the 28 samples collected from Appanoose County (Table 1 and *SI Appendix*, Table S1). In contrast, none of the nine samples collected from Black Hawk County were positive, nor were the six RPLN samples from Henry County. While the exact reasons for this heterogeneity in PCR positive response rates are unknown, this may be because of low sample size and lack of power. Alternatively, the timing of collection in relation to the SARS-CoV-2 spread in deer may play a role. For instance, the samples from Henry County were collected in April and May of 2020 during the early stages of the pandemic, well before the first positive sample was identified in deer. Similarly, all nine RPLN samples tested from Black Hawk County were collected prior to the mid-November peak of reported SARS-CoV-2 cases in humans in Iowa. Together, these results suggest the widespread presence of SARS-CoV-2 RNA in deer across Iowa, with strong evidence of temporal clustering.

### Whole Genome Sequence–Based Phylogeographic and Phylogenomic Analyses Provide Evidence of Multiple Reverse Zoonotic Spillover Events of SARS-CoV-2 to Deer and Deer-to-Deer Transmission.

To begin to understand the genomic diversity of SARS-CoV-2 associated with free-living and captive deer, we characterized the complete SARS-CoV-2 genomes from all 94 deer RPLN positive for the presence of viral RNA. A high level of sequencing coverage was obtained, and Pangolin version 3.1.11 (14) was used to identify SARS-CoV-2 lineages using previously described genome sequence analysis pipelines (15–17). Next, we used an automated vSNP pipeline (18) to identify single-nucleotide polymorphisms (SNPs) and construct phylogenetic trees in the context of 84 additional publicly available animal origin SARS-CoV-2 isolates as well as from 372 SARS-CoV-2 isolates identified from humans in Iowa during this same period (*SI Appendix*, Table S2). All newly sequenced SARS-CoV-2 consensus genomes from deer RPLN are deposited in Global Initiative on Sharing Avian Influenza Data (GISAID), and raw reads are submitted to the National Center for Biotechnology Information (NCBI) Short Read Archive (BioProject Number PRJNA776532).

The analysis identified a total of 12 SARS-CoV-2 lineages among the 94 samples from deer, with two lineages, B.1.2 (*n* = 51; 54.5%), and B.1.311 (*n* = 19; 20%), representing ∼75% of all samples (Table 1). Together with the next two most abundant lineages, B.1 (*n* = 7) and B.1.234 (*n* = 6), these four lineages represented ∼88% of SARS-CoV-2 circulating among geographically widely distributed deer in the state (Table 1; *SI Appendix*, Table S2). While the number of SARS-CoV-2 sequences from Iowa is not very high (only 372 sequences are available) during this period in publicly available data, it is noteworthy that the B.1.2 was also the most abundant (∼43.5%) SARS-CoV-2 lineage circulating in humans in Iowa (*SI Appendix*, Table S2). In contrast, the B.1.311 lineage, accounting for about one-fifth of the isolates in deer, was relatively poorly represented (∼1.6%) among the publicly available SARS-CoV-2 from humans in Iowa (*SI Appendix*, Table S2). The second-most abundant SARS-CoV-2 lineage in humans, B.1.565, accounting for ∼8.1% of available sequences, was not identified among the sampled deer. However, given the lack of representativeness of the sampling from both humans and deer, we urge caution in interpreting these findings of apparent differences in the prevalence of SARS-CoV-2 lineages between deer and sympatric human hosts.

The temporal and geographic patterns of clustering of SARS-CoV-2 lineages, together with phylogenetic analyses, provide strong evidence of multiple likely zooanthroponotic spillovers from humans to deer. This is evidenced by the near-simultaneous recovery of multiple SARS-CoV-2 lineages within temporally and geographically restricted deer herds at various locations throughout the state (*SI Appendix*, Tables S1 and S2). For instance, between April 2020 and December 2020, 22 of 23 SAR-CoV-2 positive samples recovered from deer in a game preserve in southeastern Iowa were of B.1.2 lineage. However, the single outlier isolate lineage B.1.311 was recovered from a deer at this preserve together with 12 specimens harboring the B.1.2 lineage on the same day (24 November 2020). Similarly, in the Yellow River State Forest area in Allamakee County, during the 5 d between 5 December and 9 December, all 11 hunter-killed deer were positive for the presence of SARS-CoV-2 RNA in their RPLNs. Nine of these samples were represented by the B.1.2 lineage. The two outliers, represented by B.1.311 and B.1.459 lineages were recovered from hunter-killed deer on the same date, 8 December 2020, as was another deer harboring the B.1.2 lineage. All these deer were hunted within a few miles of each other. A third example of the near-synchronous recovery of genetically distinctive SARS-CoV-2 lineages from infected deer is from the Volga River State Recreation Area in Fayette County, where four lineages were recovered from the RPLN of hunter-killed deer within a 2-mi radius in the 3 d spanning 7 December through 9 December 2020. A final example is from within a 5-mi radius of the Lake Rathbun area of Appanoose County where, on a single day, 10 positive deer RPLNs were harvested by hunters. Five distinct lineages were represented among these samples—lineage B.1.311 was recovered from six deer RPLNs, while lineages B.1.362, B.1.240, B.1.400, and B.1 were recovered from one RPLN each. Together, these findings strongly suggest multiple point sources of spillover of distinct SARS-CoV-2 lineages to captive and free-living deer.

Recent evidence suggests that experimentally infected fawns are readily infected and transmit the virus to other susceptible fawns between 3 d and 5 d postinfection. The virus can be recovered from the palatine tonsils and RPLNs of infected animals for up to 21 d postexposure (6). Similarly, it has been shown that adult white-tailed deer are also highly susceptible to SARS-CoV-2 infection and transmit the virus through direct contact and vertically from doe to fetus (12). However, evidence for deer-to-deer transmission of SARS-CoV-2 in free-living deer was not well documented. Deer experimentally infected with SARS-CoV-2 show no notable clinical signs; however, pathological changes characterized by rhinitis, marked attenuation of the respiratory epithelium of the trachea, bronchitis, and, in some cases, bronchiolitis were observed (12). The clinical outcomes of SARS-CoV-2 infection in free-living deer and whether or not they develop symptoms or disease remain unknown. This is important, since nearly three-quarters of humans who test positive for SARS-CoV-2 by RT-PCR also remain asymptomatic (19). Hence, well-powered longitudinal studies may be needed to get sufficient statistical power to determine the nature of the clinical outcome of SARS-CoV-2 infection in deer.

To explore evidence for potential sylvatic transmission in free-living deer, we applied a molecular epidemiologic approach to explore the temporal patterns of recovery of SARS-CoV-2 lineages from free-living deer to identify possible evidence of deer-to-deer transmission. One example is evident from the Lake Rathbun area of Appanoose County, where lineage B.1.311 was predominant among deer, representing 14 of 21 positive samples (*SI Appendix*, Tables S1 and S2). The first RPLN sample harboring a B.1.311 lineage was recovered on 5 December 2020, from a hunter-killed deer from this area. This was followed by additional recoveries of B.1.311 on 8 December 2020, and then again on 2 January 2021, and 9 January 2021—more than a month apart—and with high viral loads in the lymph nodes suggestive of active infection. Together with the observation that the B.1.311 lineage was less frequently reported (based on available sequences) from humans in Iowa as well as in deer in other Iowa counties, the results suggest the continued circulation of this lineage among deer in free-living settings. However, it is important to note that, in the absence of a comprehensive longitudinal study of circulating lineages, it is not possible to formally exclude the possibility that B.1.311 was circulating within humans or other hosts in this area, and the deer were repeatedly exposed to the same point source(s) of infection.

**Fig. 3.**
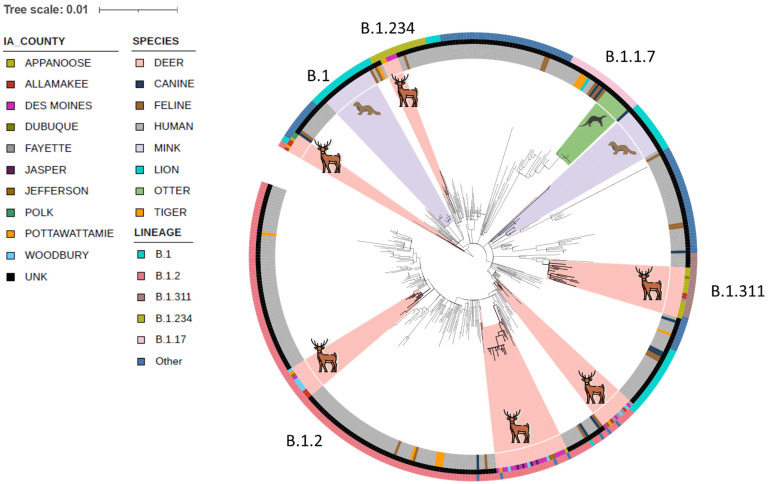
Whole-genome SNP-based phylogenies of 94 SARS-CoV-2 genomes recovered from free-living and captive white-tailed deer in Iowa. Whole genome sequences of all 94 SARS-CoV-2 positive samples from RPLNs were analyzed in the context of 92 publicly available genomes from animal origin SARS-CoV-2 isolates and 312 human SARS-CoV-2 genomes circulating in Iowa during this same period (*SI Appendix*, Table S3). The genome sequences were screened for quality, SNP positions called against the SARS-CoV-2 reference genome (NC_045512), and SNP alignments used to generate a maximum-likelihood phylogenetic tree using RAxML. The results show several genetically distinct clusters of animal and human SARS-CoV-2 lineages circulating within Iowa white-tailed deer, with clades highlighted in salmon color, consistent with multiple spillover events from humans to deer. Six branches with shared human and white-tailed deer SARS-CoV-2 isolates circulating in Iowa are highlighted. The genome sequences from white-tailed deer were genetically distinct from isolates from outbreaks in farmed mink (periwinkle color) and otters (fern color) but were closely related to SARS-CoV-2 genomes recovered from humans in Iowa.

Finally, to better visualize phylogenetic relationships among circulating SARS-CoV-2 originating in free-living and captive deer, we generated an SNP-based maximum likelihood tree including available human and animal lineage isolates (Fig. 3 and *SI Appendix*, Table S3). As evident from the branching patterns of the phylogram, the results highlight the presence of multiple independent but closely related SARS-CoV-2 lineages circulating among deer in Iowa, as well as providing strong evidence for transmission within deer, as many of the genomes from individual deer shared complete genomic identity (no SNPs) or differed by between one and five SNPs. The results also highlight several branches with shared human and deer origin SARS-CoV-2 isolates circulating in Iowa that are related to but distinct from isolates previously identified from outbreaks from animals such as farmed mink or otters or other domesticated animal species. Hence, taken together, the results provide strong evidence of multiple spillover events of SARS-CoV-2 and the subsequent circulation of these strains within free-living and captive deer.

### Broader Implications for the Ecology of SARS-CoV-2.

Most viruses causing disease in humans have originated in animals, and many are capable of transmitting between multiple host species (20, 21). The ability to infect a range of host species is a risk factor for disease emergence (22, 23). Despite this knowledge, reservoir hosts are rarely identified and studied. Indeed, the wild animal reservoirs of SARS-1, SARS-CoV-2, and Middle East respiratory syndrome (MERS)-CoV are still not known. Indeed, while there is good evidence that dromedaries and bats may harbor MERS-CoV (24, 25), and horseshoe bats harbor SARS (26), wild animal reservoirs of SARS, SARS-CoV-2, and MERS-CoV are still not well understood. There have been numerous cases of isolated human-to-animal transmission of SARS-CoV-2 involving companion, farmed, and zoo animals since the COVID-19 pandemic began (8, 9, 27, 28). Our study provides evidence of widespread dissemination of SARS-CoV-2 into any free-living species, in this instance, white-tailed deer. While the precise routes of transmission of SARS-Cov-2 from humans to deer are unknown, there are several ways in which deer may be exposed to the virus from humans, including through feeding in backyards or even when a susceptible deer may come in contact with potentially infectious material (such as saliva, urine, etc.) from an infected human in forested areas or exurban environments. Deer may also become exposed to SARS-CoV-2 through contact with wastewater discharges, infected fomites, or other infected animals. Regardless of the route of transmission from humans, our results suggest that deer have the potential to emerge as a major reservoir host for SARS-CoV-2, a finding that has important implications for the future trajectory of the pandemic.

What might be the implications of deer emerging as reservoirs of SARS-CoV-2? When pathogens infect a single host species, the population dynamics are intrinsically unstable, and an outbreak spreads rapidly through a population and then fades out as hosts either develop immunity or die from the infection. The outbreak’s trajectory depends on the basic reproductive number (R_0_) and the generation time of the infection, but this changes when a pathogen is a generalist and infects multiple host species. In this instance, the dynamics are dominated by what occurs within reservoir hosts, defined as species which can maintain the infection and from which infection is transmitted to other hosts (29).

A reservoir host can facilitate viral evolution and the emergence of lineages with increased virulence for the original host. Since many animal species already harbor an extensive array of endemic endogenous CoVs, the presence of a free-living reservoir host for SARS-CoV-2 may provide an opportunity for the virus to recombine and acquire or evolve increased fitness traits such as increased virulence, transmissibility, pathogenicity, and immune evasion (30). Evidence of some of these exists in the Denmark mink spillover event where the Y435F substitution (which conferred increased affinity of the spike protein to human ACE-2) evolved after human-to-mink transmission (8, 10, 30). Animal reservoirs can also provide a refuge outside of a largely immune/vaccinated human population and thus increase the threat of subsequent reemergence of ancestral genotypes into immunologically naïve or susceptible human hosts (31). Such was the case with the 2009 A-H1N1 (swine flu) pandemic where the virus, related to both the pandemic 1918 strain and strains circulating in the early 20th century, spilled over to humans from infected pigs (31, 32).

Predicting how a virus using a new host species can affect virulence in the primary host is not simple. In theory, pathogen evolution within a single-host system involves trade-offs with potential for transmission, but this becomes more complicated in multiple-host systems (33). With the infection spreading so quickly through the deer population, as seen in our study, this could potentially result in fadeout, with insufficient susceptible deer recruits to sustain the infection within the deer population alone. Alternatively, with sizeable annual birth cohorts or invasion into areas where deer have not previously been infected, the virus may continue to spread among susceptible deer or circulate within the deer population. However, even while the dynamics in these multihost systems can be complex, they often result in more stable dynamics with multiple reservoir hosts, and pathogens that utilize many hosts can be at a selective advantage, since they are not lost in case they fade out in an individual species.

### Study Limitations.

The study has several limitations. The RPLN samples tested were from only one state in the United States, and the sampling was not uniform within the state. However, while the generalizability of our findings remains to be tested, we see no reason why this scenario has also not already played out in other regions with large deer populations with opportunities for contact with humans. Another limitation of our study was that RPLN samples tested were all from 2020 and early 2021, representing the early part of the pandemic before the global dissemination of the highly successful Alpha and Delta variants. Hence, surveillance efforts with robust longitudinal sampling approaches are urgently needed to determine whether deer will become long-term reservoirs for SARS-CoV-2 and potentially assume a role as generators of novel variant viruses that may repeatedly reemerge in humans or spill over to other animal hosts.

### Concluding Comments and Future Directions.

White-tailed deer are the most abundant wild cervid species in the United States, with an estimated 25 million individuals, and deer hunting is the most popular form of hunting in the United States, contributing over $20 billion to the US GDP and supporting more than 300,000 jobs in 2016 (34). In addition to the abundance, social relevance, and economic importance of deer to the US economy, the discovery of sylvatic and enzootic transmission in a substantial fraction of free-living deer has important implications for the natural ecology and long-term persistence of SARS-CoV-2, including through spillover to other free-living or captive animals and potential for spillback to humans.

In this context, there are several important questions that require urgent attention. For instance, it is important to understand whether the observed widespread SARS-CoV-2 infection and transmission among white-tailed deer will result in virus adaptation to cervids similar to what has been observed in other species such as the mustelids. Previously, a high similarity in the white-tailed deer ACE-2 sequence was shown relative to human ACE-2, and the changes within the domains that bind the SARS-CoV-2 Spike protein are at residues D30E, L79M, M82T, and E329D (5, 35). Therefore, it is not altogether surprising that the deer ACE-2 receptors enable efficient binding by the Spike protein for viral entry, perhaps even slightly better than humans (35), that will allow the highly efficient transmission of SARS-CoV-2 in deer. Hence, it is tempting to predict that, unlike the observed rapid adaptions in Spike in mustelids with a restricted ACE-2 receptor, any adaptations of SARS-CoV-2 in deer during the initial waves of infection are more likely to occur in regions outside of the Spike protein. However, since experimental SARS-CoV-2 infection in deer elicits a rapid neutralizing antibody immune response (12), future investigations should closely monitor the evolutionary trajectories of SARS-CoV-2, using robust surveillance and whole genome sequencing to better understand the drivers and mechanisms of adaption of SARS-CoV-2 in a free-living natural reservoir host.

Given their abundance and widespread range in North, Central, and South America, it is important to consider that white-tailed deer overlap with a number of other wild and domestic species that are either known to be susceptible or predicted to be susceptible [i.e., deer mice, bushy-tailed woodrats, and house cats (36)]. Hence, it will be important to understand and monitor whether the high levels of infection in deer observed in our studies might result in spillover and onward transmission to other hosts, with opportunities for further viral adaptation. In particular, we note that domesticated cattle have shown low susceptibility to infection with low levels of viral replication and only limited seroconversion when experimentally challenged with extant lineages of SARS-CoV-2 (36–38). However, it remains unknown whether cattle and other large, farmed ungulate species, including sheep, goats, camelids, or equids, may become susceptible to emergent SARS-CoV-2 variants adapted in deer. Hence, it will be important to establish robust and comprehensive surveillance programs to monitor the evolutionary trajectories of SARS-CoV-2 in white-tailed deer together with potential spillover to other susceptible peridomestic and domestic animals.

Finally, our findings highlight that, to predict or prevent the emergence of the next pandemic and control infectious diseases with pandemic and panzootic potential, a better understanding of the human–animal molecular and ecological interface and its relevance to infection transmission dynamics is essential (9). Thus, we identify an urgent need to implement a more proactive and robust “One Health” approach to better understand the ecology and evolution of SARS-CoV-2 in deer and other animal species, including through active surveillance and longitudinal studies.

## Materials and Methods

### RPLN Samples.

The Iowa Department of Natural Resources (DNR) routinely collects medial RPLNs from white-tailed deer statewide for use in its Chronic Wasting Disease (CWD) surveillance program. Paired RPLNs were collected by trained field staff or partners (e.g., taxidermists), placed into separate Whirl-Pak bags, and frozen at −20 °F. Individuals involved in sample collection wore personal protective equipment, consisting of disposable gloves at a minimum. While masking was not required, the Iowa DNR had mandatory quarantine protocols in place for all staff that were symptomatic or may have been exposed. A total of 283 RPLN samples collected between April 2020 to January 2021 were studied (*SI Appendix*, Table S1). An additional 60 RPLN archived samples from the 2019/2020 CWD surveillance season (i.e., before the SARS-CoV-2 pandemic started) were included as negative control samples (*SI Appendix*, Table S1).

### RNA Extraction.

RNA was extracted from RPLNs by adding 3 mL of Universal Transport Medium (UTM, Copan) to a Whirl-Pak bag containing the tissue and placing the bag in the stomacher on a high setting for 120 s. Liquid was recovered and centrifuged at 3,000 rpm for 5 min to pellet cellular debris. Then 400 μL of the RPLN tissue homogenate supernatant was used for viral RNA extraction with a KingFisher Flex machine (ThermoFisher Scientific) with the MagMAX Viral/Pathogen extraction kit (ThermoFisher Scientific) following the manufacturer’s instructions.

### Detection of SARS-CoV-2 Viral RNA by RT-PCR.

The presence of SARS-CoV-2 nucleic acid was assessed by a real-time RT-PCR assay using the OPTI Medical SARS-CoV-2 RT-PCR kit following the manufacturer’s instructions on an ABI 7500 Fast instrument (ThermoFisher Scientific). The OPTI Medical SARS-CoV-2 RT-PCR assay detects two different targets in the gene encoding viral nucleocapsid (N) protein coding region (13, 39). The assay is highly sensitive, with a limit of detection of 0.36 copies per µL. The internal control Rnase P (RP) was used to ensure samples were not inadvertently contaminated with human origin tissue or fluids during harvesting or processing. All samples were tested and found negative for the presence of the human RP gene by RT-PCR. We generated a standard curve using SARS-CoV-2 RNA with a known copy number. Using the standard curve, viral RNA copies per milliliter of tissue homogenate were calculated.

To ensure assay specificity, a subset of 25 positive and 25 negative samples were additionally tested with the TaqPath kit (ThermoFisher Scientific) that targets the SARS-CoV-2 ORF1ab, N gene, and S gene (39, 40). All results were concordant with both assays. As an additional check of assay specificity, all 60 RPLN samples collected in 2019 prior to the first reported SARS-CoV-2 case in humans in the United States were negative for the presence of SARS-CoV-2 RNA.

### SARS-CoV-2 Genome Sequencing.

Total RNA extracted from RPLN samples was used for sequencing the whole genomes of SARS-CoV-2 as previously described (14–16, 41). Briefly, sequencing libraries were prepared according to version 4 of the ARTIC nCoV-2019 protocol (42). We used a semiautomated workflow that employed BioMek i7 liquid-handling workstations (Beckman Coulter Life Sciences) and MANTIS automated liquid handlers (FORMULATRIX). Short sequence reads were generated with a NovaSeq 6000 instrument (Illumina). To ensure a very high depth of coverage, the RPLN sequencing libraries were prepared in duplicate and sequenced with an SP 300 cycle reagent kit.

### SARS-CoV-2 Genome Sequence Analysis and Identification of Variants.

Viral genomes were assembled with the BV-BRC SARS-Cov2 assembly service (43, 44). The BV-BRC assembly service currently uses a pipeline that is similar to the One Codex SARS-CoV-2 variant-calling pipeline (45). Briefly, the pipeline uses seqtk version 1.3-r116 for sequence trimming (46), minimap version 2.1 (47) for aligning reads against reference genome Wuhan-Hu-1 (ref. 48, NC_045512.2; ref. 49), samtools version 1.11 (50) for sequence and file manipulation (51), and iVar version 1.2.2 (52) for primer trimming and variant calling (53). To increase stringency, the minimum read depth for assemblies (based on samtools mpileup) was set at three to determine consensus. Genetic lineages, variants being monitored, and variants of concern were identified and designated by Pangolin version 3.1.11 (14) with pangoLEARN module 2021-08-024 . SNPs were identified using the vSNP (18) SNP analysis program.

QGIS (geographic information system) mapping software version 3.16.10 was used to visually portray the geographic location of the white-tailed deer sampled (54).

## Supplementary Material

Supplementary File

## Data Availability

All SARS-CoV-2 consensus genomes are deposited in GISAID, https://www.gisaid.org/; Accession numbers EPI_ISL_5804716–EPI_ISL_5804787 and EPI_ISL_6425266–EPI_ISL_6425287, and raw reads have been submitted to NCBI’s Short Read Archive (BioProject Number: PRJNA776532).
