# Supplementary material for: Multiple spillovers from humans and onward transmission of SARS-CoV-2 in white-tailed deer
Source: Proc Natl Acad Sci U S A. 2022 Jan 25;119(6):e2121644119. doi: 10.1073/pnas.2121644119 (PMC8833191; doi:10.1073/pnas.2121644119)
Supplement: Supplementary File [file pnas.2121644119.sapp.pdf]

PNAS
www.pnas.org

Supplementary Information for
Multiple spillovers from humans and onward transmission of SARS-CoV-2 in white-tailed deer

Suresh V. Kuchipudi,[a*] Meera Surendran-Nair[a], Rachel M. Ruden[b,c], Michele Yon[d], Ruth H. Nissly[a], Kurt J. Vandegrift[e], Rahul K. Nelli[c], Lingling Li[d], Bhushan M. Jayarao[d], Costas D. Maranas[f], Nicole Levine[g], Katriina Willgert[h], Andrew J. K. Conlan[h], Randall J. Olsen[i,j], James J. Davis[k], James M. Musser[i,j], Peter J. Hudson[e], and Vivek Kapur[g*].

*Correspondence to: Suresh V. Kuchipudi and Vivek Kapur, **Email:** skuchipudi@psu.edu and vkapur@psu.edu

This PDF file includes:

Figures S1 to S2
Tables S1 to S3

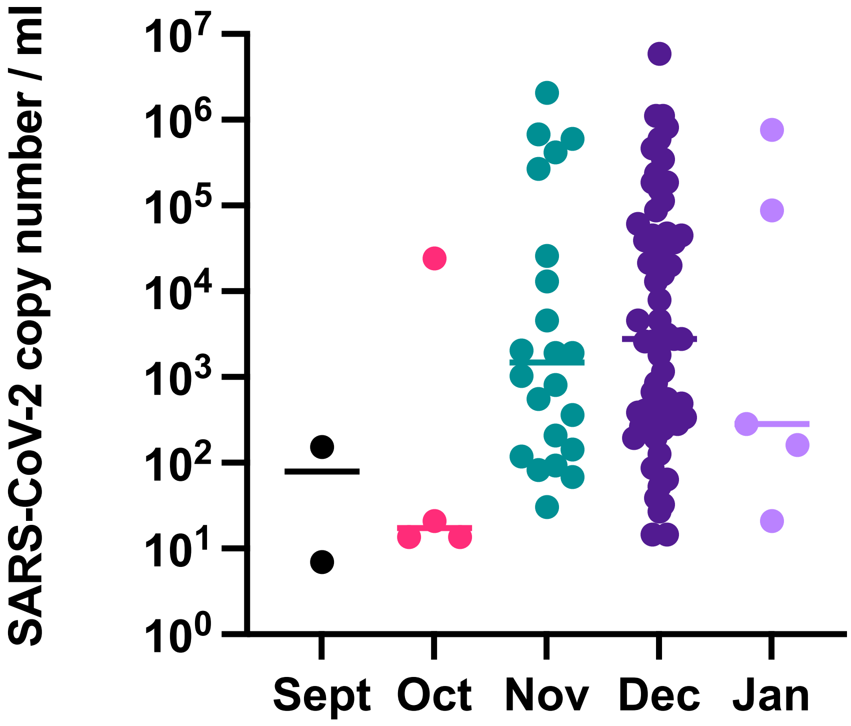

Fig. S1. Temporal changes in distribution of SARS-CoV-2 viral genome copy numbers in white-tailed deer RPLNs. As the positivity proportion among the collected samples increased over the months of collection depicted on X axis (Sept 2020 to Jan 2021), the viral copy numbers (y-axis) increased in a range of 6.9 to 5.9 x 10^6 copies/ml with a median value of 1.9 x 10^3 copies/ml.

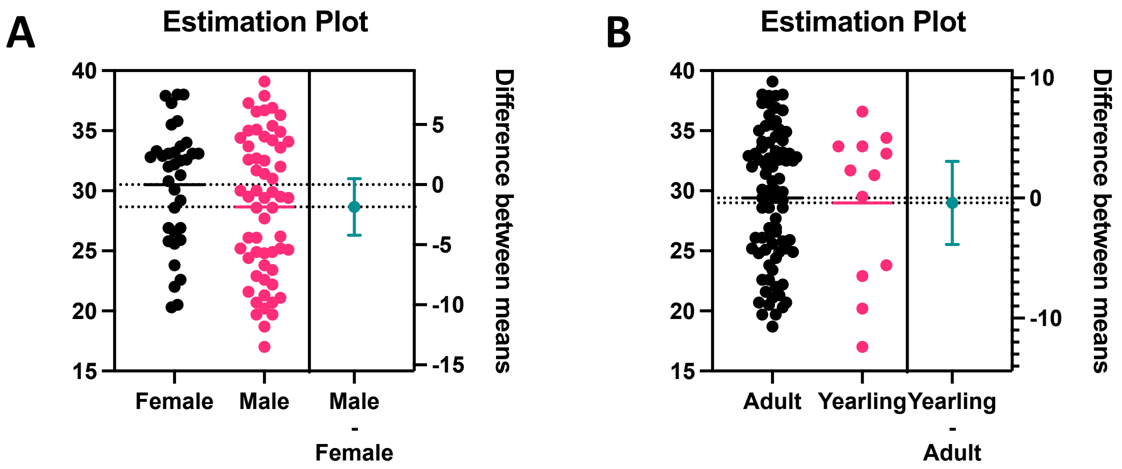

Fig. S2. Estimation plots of gender and age associated effects on the distribution of Ct values from RPLN SARS-CoV-2 positive samples. The results show no significant difference based on Sex (**A**) or Age (**B**) in proportion of SARS-CoV-2 positive samples

Table S1. Sample metadata.

Samples Included

Sample_ID	Date Collected	Source	Age	Sex	County	Habitiat	Rt-PCR Ct value	SARSCoV-2 Lineage
207162	4/8/20	Roadkill	Yearling	Female	Woodbury	Free living	NEG	
207159	4/13/20	Roadkill	Adult	Female	Woodbury	Free living	NEG	
207160	4/13/20	Roadkill	Adult	Female	Woodbury	Free living	NEG	
208000	4/28/20	Preserve	Yearling	Female	SE Iowa	Captive	NEG	
208001	4/28/20	Preserve	Fawn	Male	SE Iowa	Captive	NEG	
208002	4/28/20	Preserve	Yearling	Female	SE Iowa	Captive	NEG	
208003	4/28/20	Preserve	Adult	Female	SE Iowa	Captive	NEG	
207143	5/12/20	Roadkill	Adult	Female	Woodbury	Free living	NEG	
208004	5/13/20	Preserve	Adult	Male	SE Iowa	Captive	NEG	
208005	5/13/20	Preserve	Adult	Female	SE Iowa	Captive	NEG	
203001	5/21/20	Roadkill	Yearling	Male	Black Hawk	Free living	NEG	
207177	6/19/20	Roadkill	Adult	Male	Woodbury	Free living	NEG	
207183	6/19/20	Roadkill	Yearling	Female	Woodbury	Free living	NEG	
207185	6/19/20	Roadkill	Adult	Female	Woodbury	Free living	NEG	
207186	6/19/20	Roadkill	Adult	Female	Woodbury	Free living	NEG	
206518	8/19/20	Roadkill	Adult	Female	Pottawattamie	Free living	NEG	
208300	8/21/20	Preserve	Adult	Male	NE Iowa	Captive	NEG	
206519	9/10/20	Roadkill	Adult	Male	Pottawattamie	Free living	NEG	
208006	9/18/20	Preserve	Adult	Male	SE Iowa	Captive	NEG	
208007	9/18/20	Preserve	Adult	Male	SE Iowa	Captive	NEG	
208008	9/18/20	Preserve	Adult	Male	SE Iowa	Captive	NEG	
208009	9/18/20	Preserve	Adult	Male	SE Iowa	Captive	NEG	
208010	9/18/20	Preserve	Adult	Male	SE Iowa	Captive	NEG	
208011	9/18/20	Preserve	Adult	Male	SE Iowa	Captive	NEG	
208012	9/18/20	Preserve	Adult	Female	SE Iowa	Captive	NEG	
206520	9/20/20	Target/Sick	Adult	Male	Pottawattamie	Free living	NEG	
207144	9/20/20	Target/Sick	Adult	Female	Woodbury	Free living	NEG	
207233	9/21/20	Hunter Killed	Adult	Female	Woodbury	Free living	NEG	
207234	9/22/20	Hunter Killed	Adult	Female	Woodbury	Free living	NEG	
207235	9/22/20	Hunter Killed	Yearling	Female	Woodbury	Free living	NEG	
207236	9/22/20	Hunter Killed	Adult	Female	Woodbury	Free living	NEG	
207237	9/22/20	Hunter Killed	Adult	Female	Woodbury	Free living	NEG	
207238	9/22/20	Hunter Killed	Adult	Female	Woodbury	Free living	NEG	
206521	9/23/20	Roadkill	Adult	Female	Pottawattamie	Free living	NEG	
206522	9/24/20	Roadkill	Adult	Female	Pottawattamie	Free living	NEG	
206523	9/24/20	Roadkill	Adult	Male	Pottawattamie	Free living	NEG	
203033	9/25/20	Hunter Killed	Adult	Female	Dubuque	Free living	NEG	
203034	9/25/20	Hunter Killed	Yearling	Female	Dubuque	Free living	NEG	
207239	9/26/20	Hunter Killed	Adult	Female	Woodbury	Free living	NEG	
207240	9/26/20	Hunter Killed	Adult	Female	Woodbury	Free living	NEG	
208013	9/28/20	Preserve	Adult	Male	SE Iowa	Captive	NEG	
208014	9/28/20	Preserve	Adult	Female	SE Iowa	Captive	NEG	
208015	9/28/20	Preserve	Adult	Male	SE Iowa	Captive	NEG	
208016	9/28/20	Preserve	Adult	Male	SE Iowa	Captive	NEG	
208017	9/28/20	Preserve	Adult	Male	SE Iowa	Captive	39.1	B.1.2
208018	9/28/20	Preserve	Adult	Male	SE Iowa	Captive	NEG	
208019	9/28/20	Preserve	Adult	Male	SE Iowa	Captive	NEG	
208020	9/28/20	Preserve	Adult	Male	SE Iowa	Captive	NEG	
208021	9/28/20	Preserve	Adult	Male	SE Iowa	Captive	NEG	
208022	9/28/20	Preserve	Adult	Male	SE Iowa	Captive	NEG	

Sample_ID	Date Collected	Source	Age	Sex	County	Habitiat	Rt-PCR Ct value	SARSCoV-2 Lineage
208023	9/28/20	Preserve	Adult	Male	SE Iowa	Captive	NEG	
208024	9/28/20	Preserve	Adult	Male	SE Iowa	Captive	NEG	
208025	9/28/20	Preserve	Adult	Male	SE Iowa	Captive	NEG	
207241	9/29/20	Hunter Killed	Adult	Female	Woodbury	Free living	NEG	
207242	9/29/20	Hunter Killed	Adult	Female	Woodbury	Free living	NEG	
207337	9/30/20	Roadkill	Adult	Male	Woodbury	Free living	34.1	B.1.2
207243	10/5/20	Hunter Killed	Adult	Female	Woodbury	Free living	NEG	
207244	10/5/20	Hunter Killed	Adult	Female	Woodbury	Free living	NEG	
208026	10/8/20	Preserve	Adult	Male	SE Iowa	Captive	NEG	
208027	10/8/20	Preserve	Adult	Male	SE Iowa	Captive	NEG	
208028	10/8/20	Preserve	Adult	Male	SE Iowa	Captive	NEG	
208029	10/8/20	Preserve	Adult	Male	SE Iowa	Captive	NEG	
208030	10/8/20	Preserve	Adult	Male	SE Iowa	Captive	NEG	
208031	10/8/20	Preserve	Adult	Male	SE Iowa	Captive	NEG	
208032	10/8/20	Preserve	Adult	Male	SE Iowa	Captive	NEG	
208033	10/8/20	Preserve	Adult	Male	SE Iowa	Captive	NEG	
208034	10/8/20	Preserve	Adult	Male	SE Iowa	Captive	NEG	
208035	10/8/20	Preserve	Adult	Male	SE Iowa	Captive	NEG	
208036	10/8/20	Preserve	Adult	Male	SE Iowa	Captive	NEG	
208037	10/8/20	Preserve	Adult	Male	SE Iowa	Captive	NEG	
208038	10/8/20	Preserve	Adult	Male	SE Iowa	Captive	NEG	
208039	10/8/20	Preserve	Adult	Male	SE Iowa	Captive	NEG	
208040	10/8/20	Preserve	Adult	Male	SE Iowa	Captive	NEG	
208041	10/8/20	Preserve	Adult	Male	SE Iowa	Captive	NEG	
208042	10/8/20	Preserve	Adult	Male	SE Iowa	Captive	NEG	
208043	10/8/20	Preserve	Adult	Female	SE Iowa	Captive	38	B.1.2
207245	10/9/20	Hunter Killed	Adult	Female	Woodbury	Free living	NEG	
207349	10/11/20	Roadkill	Adult	Female	Woodbury	Free living	NEG	
206524	10/12/20	Roadkill	Adult	Female	Pottawattamie	Free living	NEG	
202276	10/13/20	Hunter Killed	Adult	Female	Black Hawk	Free living	NEG	
208044	10/13/20	Preserve	Adult	Female	SE Iowa	Captive	NEG	
208045	10/13/20	Preserve	Adult	Male	SE Iowa	Captive	NEG	
208046	10/13/20	Preserve	Adult	Male	SE Iowa	Captive	NEG	
208047	10/13/20	Preserve	Adult	Male	SE Iowa	Captive	NEG	
208048	10/13/20	Preserve	Adult	Male	SE Iowa	Captive	NEG	
208049	10/13/20	Preserve	Adult	Male	SE Iowa	Captive	NEG	
208050	10/13/20	Preserve	Adult	Male	SE Iowa	Captive	NEG	
208051	10/13/20	Preserve	Adult	Male	SE Iowa	Captive	NEG	
208052	10/13/20	Preserve	Adult	Male	SE Iowa	Captive	NEG	
206525	10/13/20	Roadkill	Adult	Male	Pottawattamie	Free living	NEG	
207246	10/13/20	Target/Sick	Adult	Female	Woodbury	Free living	NEG	
202277	10/14/20	Hunter Killed	Yearling	Female	Black Hawk	Free living	NEG	
206526	10/14/20	Target/Sick	Adult	Female	Pottawattamie	Free living	NEG	
206527	10/15/20	Roadkill	Adult	Female	Pottawattamie	Free living	38	B.1.2
207385	10/15/20	Roadkill	Adult	Female	Woodbury	Free living	NEG	
202278	10/16/20	Hunter Killed	Yearling	Female	Black Hawk	Free living	NEG	
202275	10/17/20	Hunter Killed	Yearling	Female	Black Hawk	Free living	NEG	
208325	10/17/20	Target/Escape	Adult	Male	Washington	Captive	NEG	
208383	10/19/20	Target/Escape	Adult	Male	Keokuk	Captive	NEG	
207247	10/21/20	Hunter Killed	Adult	Female	Woodbury	Free living	NEG	
207248	10/21/20	Roadkill	Adult	Female	Woodbury	Free living	NEG	
208053	10/22/20	Preserve	Adult	Male	SE Iowa	Captive	NEG	

Sample_ID	Date Collected	Source	Age	Sex	County	Habitiat	Rt-PCR Ct value	SARSCoV-2 Lineage
208054	10/22/20	Preserve	Adult	Male	SE Iowa	Captive	NEG	
208055	10/22/20	Preserve	Adult	Male	SE Iowa	Captive	NEG	
208056	10/22/20	Preserve	Adult	Male	SE Iowa	Captive	NEG	
208057	10/22/20	Preserve	Adult	Male	SE Iowa	Captive	NEG	
208058	10/22/20	Preserve	Adult	Male	SE Iowa	Captive	NEG	
208059	10/22/20	Preserve	Adult	Male	SE Iowa	Captive	NEG	
208060	10/22/20	Preserve	Adult	Male	SE Iowa	Captive	NEG	
208061	10/22/20	Preserve	Adult	Male	SE Iowa	Captive	NEG	
208062	10/22/20	Preserve	Adult	Male	SE Iowa	Captive	NEG	
208063	10/22/20	Preserve	Adult	Male	SE Iowa	Captive	NEG	
208064	10/22/20	Preserve	Adult	Male	SE Iowa	Captive	NEG	
208065	10/22/20	Preserve	Adult	Male	SE Iowa	Captive	NEG	
208066	10/22/20	Preserve	Adult	Male	SE Iowa	Captive	NEG	
208068	10/22/20	Preserve	Adult	Male	SE Iowa	Captive	NEG	
200334	10/27/20	Hunter Killed	Adult	Male	Appanoose	Free living	NEG	
206528	10/27/20	Roadkill	Adult	Female	Pottawattamie	Free living	NEG	
208326	10/27/20	Target/Escape	Adult	Female	Van Buren	Captive	NEG	
207219	10/27/20	Roadkill	Yearling	Female	Woodbury	Free living	NEG	
207249	10/27/20	Hunter Killed	Adult	Female	Woodbury	Free living	NEG	
207431	10/28/20	Roadkill	Adult	Female	Woodbury	Free living	25.9	B.1.2
206529	10/29/20	Roadkill	Adult	Male	Pottawattamie	Free living	NEG	
207149	10/29/20	Hunter Killed	Adult	Male	Woodbury	Free living	NEG	
200284	10/30/20	Hunter Killed	Yearling	Female	Appanoose	Free living	NEG	
207437	10/31/20	Roadkill	Adult	Male	Woodbury	Free living	37.3	B.1.2
200340	11/2/20	Hunter Killed	Adult	Male	Appanoose	Free living	NEG	
207250	11/2/20	Hunter Killed	Adult	Female	Woodbury	Free living	NEG	
208069	11/3/20	Preserve	Adult	Male	SE Iowa	Captive	NEG	
208070	11/3/20	Preserve	Adult	Male	SE Iowa	Captive	NEG	
208071	11/3/20	Preserve	Adult	Male	SE Iowa	Captive	NEG	
208072	11/3/20	Preserve	Adult	Male	SE Iowa	Captive	NEG	
208073	11/3/20	Preserve	Adult	Male	SE Iowa	Captive	NEG	
208074	11/3/20	Preserve	Adult	Male	SE Iowa	Captive	NEG	
208075	11/3/20	Preserve	Adult	Male	SE Iowa	Captive	NEG	
208076	11/3/20	Preserve	Adult	Male	SE Iowa	Captive	NEG	
208077	11/3/20	Preserve	Adult	Male	SE Iowa	Captive	NEG	
208078	11/3/20	Preserve	Adult	Male	SE Iowa	Captive	NEG	
208079	11/3/20	Preserve	Adult	Male	SE Iowa	Captive	NEG	
208080	11/3/20	Preserve	Adult	Male	SE Iowa	Captive	NEG	
208081	11/3/20	Preserve	Adult	Male	SE Iowa	Captive	NEG	
208082	11/3/20	Preserve	Adult	Female	SE Iowa	Captive	NEG	
208083	11/3/20	Preserve	Adult	Female	SE Iowa	Captive	NEG	
208084	11/3/20	Preserve	Adult	Male	SE Iowa	Captive	NEG	
206530	11/3/20	Hunter Killed	Adult	Male	Pottawattamie	Free living	NEG	
207471	11/3/20	Roadkill	Yearling	Male	Woodbury	Free living	NEG	
203062	11/4/20	Hunter Killed	Adult	Female	Jackson	Free living	NEG	
203063	11/4/20	Hunter Killed	Adult	Female	Jackson	Free living	NEG	
203064	11/4/20	Hunter Killed	Adult	Female	Jackson	Free living	NEG	
206531	11/4/20	Hunter Killed	Adult	Female	Pottawattamie	Free living	NEG	
203132	11/5/20	Hunter Killed	Yearling	Female	Dubuque	Free living	NEG	
203134	11/5/20	Hunter Killed	Yearling	Female	Dubuque	Free living	NEG	
203136	11/5/20	Roadkill	Adult	Male	Dubuque	Free living	NEG	
203140	11/5/20	Hunter Killed	Adult	Female	Dubuque	Free living	NEG	

Sample_ID	Date Collected	Source	Age	Sex	County	Habitiat	Rt-PCR Ct value	SARSCoV-2 Lineage
206532	11/5/20	Roadkill	Adult	Female	Pottawattamie	Free living	NEG	
200349	11/6/20	Roadkill	Yearling	Male	Appanoose	Free living	NEG	
206407	11/6/20	Hunter Killed	Adult	Male	Pottawattamie	Free living	18.7	B.1.2
207524	11/6/20	Roadkill	Adult	Female	Woodbury	Free living	NEG	
207530	11/7/20	Roadkill	Adult	Female	Woodbury	Free living	22	B.1.2
207531	11/8/20	Roadkill	Adult	Female	Woodbury	Free living	25.8	B.1.2
203193	11/10/20	Hunter Killed	Adult	Male	Dubuque	Free living	NEG	
203189	11/12/20	Hunter Killed	Adult	Male	Dubuque	Free living	NEG	
206670	11/12/20	Roadkill	Yearling	Male	Pottawattamie	Free living	NEG	
202282	11/13/20	Hunter Killed	Adult	Female	Black Hawk	Free living	NEG	
202283	11/13/20	Hunter Killed	Adult	Female	Black Hawk	Free living	NEG	
202284	11/13/20	Roadkill	Adult	Female	Black Hawk	Free living	NEG	
202985	11/13/20	Hunter Killed	Adult	Male	Black Hawk	Free living	NEG	
208085	11/13/20	Preserve	Adult	Male	SE Iowa	Captive	NEG	
208086	11/13/20	Preserve	Adult	Male	SE Iowa	Captive	NEG	
208087	11/13/20	Preserve	Adult	Male	SE Iowa	Captive	NEG	
208088	11/13/20	Preserve	Adult	Male	SE Iowa	Captive	NEG	
208089	11/13/20	Preserve	Adult	Male	SE Iowa	Captive	NEG	
208090	11/13/20	Preserve	Adult	Male	SE Iowa	Captive	NEG	
208091	11/13/20	Preserve	Adult	Male	SE Iowa	Captive	NEG	
208092	11/13/20	Preserve	Adult	Male	SE Iowa	Captive	NEG	
208093	11/13/20	Preserve	Adult	Male	SE Iowa	Captive	NEG	
208094	11/13/20	Preserve	Adult	Male	SE Iowa	Captive	NEG	
208327	11/13/20	Preserve	Adult	Male	SE Iowa	Captive	NEG	
207150	11/15/20	Roadkill	Adult	Male	Woodbury	Free living	31	B.1.2
208328	11/17/20	Preserve	Adult	Male	SE Iowa	Captive	NEG	
208329	11/17/20	Preserve	Adult	Male	SE Iowa	Captive	34.5	B.1.2
208330	11/17/20	Preserve	Adult	Male	SE Iowa	Captive	35.1	B.1.2
207579	11/17/20	Roadkill	Adult	Female	Woodbury	Free living	28.6	B.1
208331	11/18/20	Preserve	Adult	Male	SE Iowa	Captive	36.7	B.1.1
200030	11/19/20	Target/Escape	Adult	Male	Polk	Captive	NEG	
208067	11/20/20	Preserve	Adult	Male	Des Moines	Captive	NEG	
203253	11/22/20	Target/Sick	Adult	Male	Dubuque	Free living	NEG	
208095	11/24/20	Preserve	Adult	Male	SE Iowa	Captive	34.9	B.1.2
208096	11/24/20	Preserve	Adult	Male	SE Iowa	Captive	NEG	
208097	11/24/20	Preserve	Adult	Male	SE Iowa	Captive	NEG	
208098	11/24/20	Preserve	Adult	Male	SE Iowa	Captive	34.2	B.1.2
208099	11/24/20	Preserve	Adult	Male	SE Iowa	Captive	32.7	B.1.2
208100	11/24/20	Preserve	Adult	Male	SE Iowa	Captive	33.6	B.1.2
208101	11/24/20	Preserve	Adult	Male	SE Iowa	Captive	35.4	B.1.2
208102	11/24/20	Preserve	Adult	Male	SE Iowa	Captive	20.7	B.1.2
208103	11/24/20	Preserve	Adult	Male	SE Iowa	Captive	31.4	B.1.2
208104	11/24/20	Preserve	Adult	Male	SE Iowa	Captive	29.9	B.1.2
208105	11/24/20	Preserve	Adult	Male	SE Iowa	Captive	32	B.1.311
208106	11/24/20	Preserve	Adult	Male	SE Iowa	Captive	30	B.1.2
208107	11/24/20	Preserve	Adult	Male	SE Iowa	Captive	21.3	B.1.2
207589	11/24/20	Hunter Killed	Adult	Female	Woodbury	Free living	20.5	B.1.2
207391	11/29/20	Target/Sick	Adult	Male	Woodbury	Free living	30	B.1.2
207253	11/30/20	Hunter Killed	Adult	Female	Woodbury	Free living	26.9	B.1.311
207770	12/2/20	Roadkill	Adult	Female	Woodbury	Free living	32.8	B.1.311
203164	12/3/20	Hunter Killed	Adult	Male	Dubuque	Free living	NEG	
203229	12/4/20	Hunter Killed	Adult	Male	Dubuque	Free living	NEG	

Sample_ID	Date Collected	Source	Age	Sex	County	Habitiat	Rt-PCR Ct value	SARSCoV-2 Lineage
204472	12/5/20	Hunter Killed	Adult	Male	Allamakee	Free living	28.6	B.1.2
204473	12/5/20	Hunter Killed	Adult	Female	Allamakee	Free living	29.2	B.1.2
204478	12/5/20	Hunter Killed	Adult	Female	Allamakee	Free living	33.1	B.1.2
200276	12/5/20	Hunter Killed	Adult	Female	Appanoose	Free living	NEG	
200277	12/5/20	Hunter Killed	Adult	Female	Appanoose	Free living	NEG	
200566	12/5/20	Hunter Killed	Adult	Male	Appanoose	Free living	19.7	B.1.311
200567	12/5/20	Hunter Killed	Adult	Female	Appanoose	Free living	32.6	B.1.311
200568	12/5/20	Hunter Killed	Adult	Male	Appanoose	Free living	26.1	B.1.311
200569	12/5/20	Hunter Killed	Adult	Female	Appanoose	Free living	30.8	B.1.311
200570	12/5/20	Hunter Killed	Adult	Male	Appanoose	Free living	19.7	B.1.311
200571	12/5/20	Hunter Killed	Adult	Male	Appanoose	Free living	22.2	B.1.362
200572	12/5/20	Hunter Killed	Adult	Male	Appanoose	Free living	25.1	B.1.240
200573	12/5/20	Hunter Killed	Adult	Female	Appanoose	Free living	32	B.1.311
200574	12/5/20	Hunter Killed	Adult	Male	Appanoose	Free living	29.4	B.1.400
200772	12/5/20	Hunter Killed	Adult	Male	Appanoose	Free living	26.1	B.1
203239	12/5/20	Hunter Killed	Adult	Male	Dubuque	Free living	32.6	B.1.2
207649	12/5/20	Hunter Killed	Adult	Male	Woodbury	Free living	28.6	B.1.596
201788	12/6/20	Hunter Killed	Yearling	Male	Polk	Free living	33.7	B.1.234
201793	12/6/20	Hunter Killed	Adult	Female	Polk	Free living	30.1	B.1.119
201794	12/6/20	Hunter Killed	Yearling	Male	Polk	Free living	36.6	B.1.234
201795	12/6/20	Hunter Killed	Adult	Female	Polk	Free living	33.2	B.1.234
201796	12/6/20	Hunter Killed	Yearling	Female	Polk	Free living	33.7	B.1.264
201797	12/6/20	Hunter Killed	Yearling	Male	Polk	Free living	34.4	B.1.400
205795	12/7/20	Hunter Killed	Adult	Female	Allamakee	Free living	33.3	B.1.2
204363	12/7/20	Hunter Killed	Adult	Male	Fayette	Free living	37.9	B.1
204364	12/7/20	Hunter Killed	Adult	Male	Fayette	Free living	36.9	B.1.2
204374	12/7/20	Hunter Killed	Yearling	Female	Fayette	Free living	NEG	
205750	12/7/20	Hunter Killed	Adult	Female	Fayette	Free living	37.9	B.1.234
205751	12/7/20	Hunter Killed	Yearling	Male	Fayette	Free living	NEG	
205752	12/7/20	Hunter Killed	Yearling	Male	Fayette	Free living	NEG	
203522	12/8/20	Hunter Killed	Adult	Female	Allamakee	Free living	35.8	B.1.2
203525	12/8/20	Hunter Killed	Yearling	Male	Allamakee	Free living	20.2	B.1.311
203526	12/8/20	Hunter Killed	Adult	Female	Allamakee	Free living	26.6	B.1.459
200191	12/8/20	Hunter Killed	Adult	Male	Appanoose	Free living	25.2	B.1.311
200192	12/8/20	Hunter Killed	Adult	Male	Appanoose	Free living	35	B.1.311
200193	12/8/20	Hunter Killed	Adult	Male	Appanoose	Free living	24.9	B.1.311
200194	12/8/20	Hunter Killed	Adult	Female	Appanoose	Free living	25.6	B.1.311
200195	12/8/20	Hunter Killed	Adult	Female	Appanoose	Free living	33.1	B.1.311
200459	12/8/20	Hunter Killed	Adult	Male	Appanoose	Free living	NEG	
205769	12/8/20	Hunter Killed	Adult	Male	Fayette	Free living	29.4	B.1.2
203700	12/9/20	Hunter Killed	Adult	Male	Allamakee	Free living	23.4	B.1.2
203701	12/9/20	Hunter Killed	Adult	Male	Allamakee	Free living	20.7	B.1.2
203704	12/9/20	Hunter Killed	Yearling	Male	Allamakee	Free living	29.5	B.1.2
203705	12/9/20	Hunter Killed	Yearling	Female	Allamakee	Free living	31.3	B.1.2
201020	12/9/20	Hunter Killed	Adult	Male	Dickinson	Free living	NEG	
204668	12/9/20	Hunter Killed	Adult	Female	Fayette	Free living	32.5	B.1.311
204669	12/9/20	Hunter Killed	Adult	Female	Fayette	Free living	32.2	B.1.234
208108	12/10/20	Preserve	Adult	Male	SE Iowa	Captive	32.5	B.1.2
208109	12/10/20	Preserve	Adult	Male	SE Iowa	Captive	26.2	B.1.2
208110	12/10/20	Preserve	Adult	Male	SE Iowa	Captive	24.9	B.1.2
208111	12/10/20	Preserve	Adult	Male	SE Iowa	Captive	24.4	B.1.2
208112	12/10/20	Preserve	Adult	Male	SE Iowa	Captive	27.7	B.1.2

Sample_ID	Date Collected	Source	Age	Sex	County	Habitiat	Rt-PCR Ct value	SARSCoV-2 Lineage
208113	12/10/20	Preserve	Adult	Male	SE Iowa	Captive	25.2	B.1.2
208114	12/10/20	Preserve	Adult	Male	SE Iowa	Captive	21.1	B.1.2
208115	12/10/20	Preserve	Adult	Male	SE Iowa	Captive	24.8	B.1.2
208116	12/10/20	Preserve	Adult	Male	SE Iowa	Captive	22.6	B.1.2
201820	12/10/20	Hunter Killed	Yearling	Male	Jasper	Free living	17	B.1
201821	12/10/20	Hunter Killed	Adult	Female	Jasper	Free living	22.6	B.1
201833	12/10/20	Hunter Killed	Yearling	Male	Jasper	Free living	23.8	B.1
201835	12/10/20	Hunter Killed	Adult	Female	Jasper	Free living	26.9	B.1
201741	12/10/20	Roadkill	Adult	Male	Webster	Free living	NEG	
207707	12/10/20	Roadkill	Yearling	Male	Woodbury	Free living	31.7	B.1.2
200069	12/15/20	Hunter Killed	Adult	Female	Appanoose	Free living	32.9	B.1.119
206739	12/15/20	Roadkill	Yearling	Male	Pottawattamie	Free living	22.9	B.1.2
208334	12/17/20	Preserve	Adult	Male	SE Iowa	Captive	36.3	B.1.2
208335	12/17/20	Preserve	Adult	Male	SE Iowa	Captive	NEG	
208336	12/17/20	Preserve	Adult	Male	SE Iowa	Captive	NEG	
206533	12/18/20	Roadkill	Adult	Female	Pottawattamie	Free living	NEG	
206534	12/18/20	Roadkill	Adult	Unknown	Pottawattamie	Free living	NEG	
206535	12/22/20	Roadkill	Adult	Male	Pottawattamie	Free living	21.6	B.1.2
208117	12/23/20	Preserve	Adult	Male	SE Iowa	Captive	29.5	B.1.2
206978	12/28/20	Roadkill	Adult	Female	Pottawattamie	Free living	35.5	B.1.2
200442	1/9/21	Hunter Killed	Adult	Female	Appanoose	Free living	37.3	B.1.311
200443	1/9/21	Hunter Killed	Adult	Female	Appanoose	Free living	23.8	B.1.311
200444	1/9/21	Hunter Killed	Yearling	Female	Appanoose	Free living	33.1	B.1.311
200862	1/9/21	Hunter Killed	Adult	Female	Appanoose	Free living	20.3	B.1.362
200866	1/9/21	Hunter Killed	Adult	Female	Appanoose	Free living	34	B.1.234

Samples Excluded*

Sample_ID	Date_Collected	Sample	Age	Sex	County	Location	Location2	Why Excluded
208332	11/19/20	Preserve	Adult	Female	SE Iowa	Other Cervid	Preserve 3	Wrong Species
208333	11/19/20	Preserve	Adult	Female	SE Iowa	Other Cervid	Preserve 3	Wrong Species
208301	Not specified	Preserve	Adult	Unknown	NE Iowa	Captive	Preserve 1	Missing Date
208302	Not specified	Preserve	Adult	Unknown	NE Iowa	Captive	Preserve 1	Missing Date
208303	Not specified	Preserve	Adult	Unknown	NE Iowa	Captive	Preserve 1	Missing Date

Pre-pandemic samples included

Sample_ID	Date_Collected	Sample	Age	Sex	County	Location	RT-PCR results
193076	4/7/19	Roadkill	Yearling	Male	Clinton	Rural	NEG
193057	9/18/19	Roadkill	Yearling	Male	Black Hawk	Rural	NEG
197229	10/14/19	Hunter Killed	Adult	Male	Crawford	Rural	NEG
193146	10/16/19	Target/Sick	Adult	Female	Clinton	Rural	NEG
193148	10/16/19	Hunter Killed	Adult	F	Clinton	Public_land	NEG
193218	11/12/19	Hunter Killed	Adult	Male	Clinton	Public_land	NEG
193293	11/14/19	Hunter Killed	Adult	Female	Scott	Rural	NEG
193298	11/15/19	Hunter Killed	Adult	Male	Johnson	Rural	NEG
193304	11/15/19	Target/Sick	Yearling	Male	Scott	Public_land	NEG
194231	11/15/19	Target/Sick	Adult	Male	Black Hawk	Public_land	NEG
198715	11/16/19	Hunter Killed	Adult	Male	Warren	Rural	NEG
193305	11/18/19	Hunter Killed	Adult	Female	Scott	Public_land	NEG
193306	11/18/19	Hunter Killed	Adult	Female	Scott	Public_land	NEG
194229	11/18/19	Roadkill	Yearling	Female	Black Hawk	Rural	NEG

Sample_ID	Date_Collected	Sample	Age	Sex	County	Location	RT-PCR results
197347	11/20/19	Roadkill	Adult	Female	Crawford	Rural	NEG
193224	11/27/19	Roadkill	Adult	Male	Scott	Public_land	NEG
193355	12/2/19	Roadkill	Adult	Male	Clinton	Rural	NEG
193356	12/2/19	Roadkill	Adult	Male	Clinton	Rural	NEG
193363	12/4/19	Roadkill	Adult	Female	Scott	Rural	NEG
197200	12/7/19	Hunter Killed	Adult	Male	Crawford	Rural	NEG
193625	12/9/19	Hunter Killed	Adult	Male	Clinton	Rural	NEG
193628	12/9/19	Hunter Killed	Adult	Male	Clinton	Rural	NEG
193670	12/9/19	Hunter Killed	Yearling	Female	Clinton	Rural	NEG
193671	12/9/19	Hunter Killed	Yearling	Male	Clinton	Rural	NEG
193672	12/9/19	Hunter Killed	Adult	Female	Clinton	Rural	NEG
193673	12/9/19	Hunter Killed	Yearling	Female	Clinton	Rural	NEG
193674	12/9/19	Hunter Killed	Adult	Female	Clinton	Rural	NEG
193648	12/10/19	Hunter Killed	Adult	Female	Clinton	Rural	NEG
193725	12/10/19	Hunter Killed	Adult	Male	Clinton	Rural	NEG
193726	12/10/19	Hunter Killed	Adult	Female	Clinton	Rural	NEG
193727	12/10/19	Hunter Killed	Adult	Female	Clinton	Rural	NEG
193728	12/10/19	Hunter Killed	Adult	Female	Clinton	Rural	NEG
194509	12/10/19	Hunter Killed	Adult	Female	Taylor	Rural	NEG
193724	12/11/19	Hunter Killed	Adult	Male	Clinton	Rural	NEG
193733	12/11/19	Hunter Killed	Adult	Male	Clinton	Rural	NEG
193734	12/11/19	Hunter Killed	Adult	Male	Clinton	Rural	NEG
193735	12/11/19	Hunter Killed	Adult	Male	Clinton	Rural	NEG
193736	12/11/19	Hunter Killed	Adult	Female	Clinton	Rural	NEG
193826	12/11/19	Hunter Killed	Yearling	Female	Clinton	Rural	NEG
193828	12/11/19	Hunter Killed	Yearling	Male	Clinton	Rural	NEG
193829	12/11/19	Hunter Killed	Adult	Female	Clinton	Rural	NEG
197592	12/11/19	Hunter Killed	Adult	Female	Ida	Public_land	NEG
194853	12/12/19	Hunter Killed	Adult	Male	Black Hawk	Public_land	NEG
198536	12/12/19	Roadkill	Adult	Female	Adair	Rural	NEG
194473	12/13/19	Roadkill	Adult	Male	Hardin	Rural	NEG
191696	12/14/19	Taxidermy	Yearling	Female	Taylor	Rural	NEG
194527	12/14/19	Roadkill	Yearling	Male	Grundy	Rural	NEG
198527	12/14/19	Hunter Killed	Adult	Male	Taylor	Rural	NEG
197519	12/15/19	Locker	Adult	Female	Cherokee	Public_land	NEG
197520	12/15/19	Locker	Yearling	Male	Cherokee	Public_land	NEG
197696	12/15/19	Locker	Adult	Male	Cherokee	Public_land	NEG
198747	12/16/19	Hunter Killed	Yearling	Female	Crawford	City_bounds	NEG
198765	12/16/19	Hunter Killed	Adult	Male	Cherokee	Public_land	NEG
198534	12/17/19	Hunter Killed	Adult	Male	Madison	Rural	NEG
198549	12/17/19	Hunter Killed	Adult	Female	Adair	Rural	NEG
198749	12/19/19	Hunter Killed	Adult	Male	Ida	Rural	NEG
198770	12/26/19	Hunter Killed	Adult	Male	Crawford	Rural	NEG
190133	12/30/19	Hunter Killed	Adult	Male	Marion	Rural	NEG
190138	1/2/20	Hunter Killed	Adult	Male	Warren	Rural	NEG
196660	1/14/20	Hunter Killed	Adult	Female	Louisa	Rural	NEG

Table S2. Distribution of SARS-CoV-2 Lineages in White-tailed deer in Iowa.

County / Region*	SARS-CoV-2 Lineage (*n*)
**Allamakee**	**11**
B.1.2	9
B.1.311	1
B.1.459	1
**Appanoose**	**21**
B.1	1
B.1.119	1
B.1.234	1
B.1.240	1
B.1.311	14
B.1.362	2
B.1.400	1
**Dubuque**	**1**
B.1.2	1
**Fayette**	**6**
B.1	1
B.1.2	2
B.1.234	2
B.1.311	1
**Jasper**	**4**
B.1	4
**Polk**	**6**
B.1.119	1
B.1.234	3
B.1.264	1
B.1.400	1
**Pottawattamie**	**5**
B.1.2	5
**SE Iowa**	**27**
B.1.1	1
B.1.2	25
B.1.311	1
**Woodbury**	**13**
B.1	1
B.1.2	9
B.1.311	2
B.1.596	1
**Grand Total**	**94**

*SARS-CoV-2 positive RPLN samples were not identified from Black Hawk, Des Moines, Dickinson, Jackson, Keokuk, NE Iowa, Van Buren, Washington, and Webster counties / regions of Iowa.

Table S3. Metadata associated with 92 animal origin SARS-CoV-2 isolates and 312 human SARS-CoV-2 genomes circulating in Iowa from April 2020 through January 2021.

Tree node ID	Host	Collection date	Lineage	Location
200069	Deer	12/15/20	B.1.119	Appanoose
200191	Deer	12/8/20	B.1.311	Appanoose
200192	Deer	12/8/20	B.1.311	Appanoose
200193	Deer	12/8/20	B.1.311	Appanoose
200194	Deer	12/8/20	B.1.311	Appanoose
200195	Deer	12/8/20	B.1.311	Appanoose
200442	Deer	1/9/21	B.1.311	Appanoose
200443	Deer	1/9/21	B.1.311	Appanoose
200444	Deer	1/9/21	B.1.311	Appanoose
200566	Deer	12/5/20	B.1.311	Appanoose
200567	Deer	12/5/20	B.1.311	Appanoose
200568	Deer	12/5/20	B.1.311	Appanoose
200569	Deer	12/5/20	B.1.311	Appanoose
200570	Deer	12/5/20	B.1.311	Appanoose
200571	Deer	12/5/20	B.1.362	Appanoose
200572	Deer	12/5/20	B.1.240	Appanoose
200573	Deer	12/5/20	B.1.311	Appanoose
200574	Deer	12/5/20	B.1.400	Appanoose
200772	Deer	12/5/20	B.1	Appanoose
200862	Deer	1/9/21	B.1.362	Appanoose
200866	Deer	1/9/21	B.1.234	Appanoose
201788	Deer	12/6/20	B.1.234	Polk
201793	Deer	12/6/20	B.1.119	Polk
201794	Deer	12/6/20	B.1.234	Polk
201795	Deer	12/6/20	B.1.234	Polk
201796	Deer	12/6/20	B.1.264	Polk
201797	Deer	12/6/20	B.1.400	Polk
201820	Deer	12/10/20	B.1	Jasper
201821	Deer	12/10/20	B.1	Jasper
201833	Deer	12/10/20	B.1	Jasper
201835	Deer	12/10/20	B.1	Jasper
203239	Deer	12/5/20	B.1.2	Dubuque
203522	Deer	12/8/20	B.1.2	Allamakee
203525	Deer	12/8/20	B.1.311	Allamakee
203526	Deer	12/8/20	B.1.459	Allamakee
203700	Deer	12/9/20	B.1.2	Allamakee
203701	Deer	12/9/20	B.1.2	Allamakee

Tree node ID	Host	Collection date	Lineage	Location
203704	Deer	12/9/20	B.1.2	Allamakee
203705	Deer	12/9/20	B.1.2	Allamakee
204363	Deer	12/7/20	B.1	Fayette
204364	Deer	12/7/20	B.1.2	Fayette
204472	Deer	12/5/20	B.1.2	Allamakee
204473	Deer	12/5/20	B.1.2	Allamakee
204478	Deer	12/5/20	B.1.2	Allamakee
204668	Deer	12/9/20	B.1.311	Fayette
204669	Deer	12/9/20	B.1.234	Fayette
205750	Deer	12/7/20	B.1.234	Fayette
205769	Deer	12/8/20	B.1.2	Fayette
206527	Deer	12/7/20	B.1.2	Allamakee
206535	Deer	11/6/20	B.1.2	Pottawattamie
206739	Deer	10/15/20	B.1.2	Pottawattamie
206978	Deer	12/22/20	B.1.2	Pottawattamie
207150	Deer	12/15/20	B.1.2	Pottawattamie
207253	Deer	12/28/20	B.1.2	Pottawattamie
207337	Deer	11/15/20	B.1.2	Woodbury
207391	Deer	11/30/20	B.1.311	Woodbury
205795	Deer	9/30/20	B.1.2	Woodbury
207431	Deer	11/29/20	B.1.2	Woodbury
206407	Deer	10/28/20	B.1.2	Woodbury
207437	Deer	10/31/20	B.1.2	Woodbury
207530	Deer	11/7/20	B.1.2	Woodbury
207531	Deer	11/8/20	B.1.2	Woodbury
207579	Deer	11/17/20	B.1	Woodbury
207589	Deer	11/24/20	B.1.2	Woodbury
207649	Deer	12/5/20	B.1.596	Woodbury
207707	Deer	12/10/20	B.1.2	Woodbury
207770	Deer	12/2/20	B.1.311	Woodbury
208017	Deer	9/28/20	B.1.2	SE Iowa
208043	Deer	10/8/20	B.1.2	SE Iowa
208095	Deer	11/24/20	B.1.2	SE Iowa
208098	Deer	11/24/20	B.1.2	SE Iowa
208099	Deer	11/24/20	B.1.2	SE Iowa
208100	Deer	11/24/20	B.1.2	SE Iowa
208101	Deer	11/24/20	B.1.2	SE Iowa
208102	Deer	11/24/20	B.1.2	SE Iowa
208103	Deer	11/24/20	B.1.2	SE Iowa

Tree node ID	Host	Collection date	Lineage	Location
208104	Deer	11/24/20	B.1.2	SE Iowa
208105	Deer	11/24/20	B.1.311	SE Iowa
208106	Deer	11/24/20	B.1.2	SE Iowa
208107	Deer	11/24/20	B.1.2	SE Iowa
208108	Deer	12/10/20	B.1.2	SE Iowa
208109	Deer	12/10/20	B.1.2	SE Iowa
208110	Deer	12/10/20	B.1.2	SE Iowa
208111	Deer	12/10/20	B.1.2	SE Iowa
208112	Deer	12/10/20	B.1.2	SE Iowa
208113	Deer	12/10/20	B.1.2	SE Iowa
208114	Deer	12/10/20	B.1.2	SE Iowa
208115	Deer	12/10/20	B.1.2	SE Iowa
208116	Deer	12/10/20	B.1.2	SE Iowa
208117	Deer	12/23/20	B.1.2	SE Iowa
208329	Deer	11/17/20	B.1.2	SE Iowa
208330	Deer	11/17/20	B.1.2	SE Iowa
208331	Deer	11/18/20	B.1.1	SE Iowa
208334	Deer	12/17/20	B.1.2	SE Iowa
20-024606-001_dog_08-11/2020_TX_np_TAMU-096	Dog	8/11/21	B.1	North America / USA / Texas
20-024799-001_dog_8-12-20_TX_op_TAMU-104	Dog	missing	missing	missing
20-024801-002_feline_8-13/2020_TX_np_TAMU-122	Cat	8/13/20	B.1.2	North America / USA / Texas
20-024806-001_feline_8-21/2020_TX_op_TAMU-146	Cat	8/21/20	B.1	North America / USA / Texas
20-024807-001_dog_10-2-20_TX_np_TAMU-149	Dog	8/21/20	B.1.576	North America / USA / Texas
20-028046-004_dog_9-14-20_TX_TAMU-173	Dog	9/14/20	B.1.2	North America / USA / Texas
20-035363-001_dog_12-7-20_KS	Dog	2020-12	B.1.2	North America / USA / Kansas
20-037287-001v_dog_12-14-20_PA	Dog	12/14/20	B.1.509	North America / USA / Pennsylvania
21-002342-001s_dog_01-15-21_FL_na	Dog	1/15/21	B.1.526	North America / USA / Florida
21-005988-002s_dog_02-12-12_TX_op_TAMU-466	Dog	2/12/21	B.1.1.7	North America / USA / Texas / Brazos County
21-007025-001s_zc	Dog	3/3/21	B.1.526	North America / USA / Connecticut
21-012417-001v_zc	Feline	4/16/21	B.1.1.7	North America / USA / Mississippi
20-026484-001_feline_8-8-20_KY	Feline	9/8/20	B.1.1.186	North America / USA / Kentucky
20-028488-001_feline_9-25-20_AL	Feline	9/25/20	B.1.234	North America / USA / Alabama
20-028752-001_feline_9-22-20_TX_TAMU-197	Feline	9/22/20	B.1.234	North America / USA / Texas
20-028754-002_feline_9-24-20_TX_TAMU-201	Feline	unknown	unknown	unknown
20-029571-001_feline_10-2-20_PA	Feline	10/2/20	B.1.369	North America / USA / Pennsylvania
20-029604-001_feline_10-6-20_TX_TAMU-212	Feline	10/6/20	B.1.2	North America / USA / Texas
20-032807-002_feline_10-22-20_TAMU-269	Feline	10/22/20	B.1	North America / USA / Texas
20-032807-007_feline_10-22-20_TAMU-270	Feline	10/22/20	B.1	North America / USA / Texas
20-035363-001v_dog_12-7-20_KS	Feline	unknown	unknown	unknown

Tree node ID	Host	Collection date	Lineage	Location
20-037760-004_feline_12-18-20_VA_tw	Feline	12/18/20	B.1.240	North America / USA / Virginia
21-000218-001s_feline_01-04-21_KS_np_original_RNA_repeat	Feline	1/4/21	B.1.2	North America / USA / Kansas
21-000296-001s_feline_12-28-20_CA_na	Feline	12/28/20	B.1	North America / USA / California
21-001379-001s_feline_01-07-21_AR_na	Feline	1/7/21	B.1.2	North America / USA / Arkansas
21-002490-001s_feline_01-23-21_CT_na	Feline	1/23/21	B.1.1.486	North America / USA / Connecticut
21-003328-001s_feline_01-26-21_FL_na	Feline	1/26/21	B.1.2	North America / USA / Florida
21-003696-001s_feline_01-30-21_CA_na	Feline	1/30/21	B.1.429	North America / USA / California
21-004025-001s_feline_01-29-21_AZ_na	Feline	1/29/21	B.1.429	North America / USA / Arizona
21-005988-005s_feline_02-12-12_TX_np_TAMU-467	Feline	2/12/21	B.1.1.7	North America / USA / Texas / Brazos County
21-007630-001s_feline_03-06-21_NJ_na	Feline	3/6/21	B.1.526	North America / USA / New Jersey
21-012714-001v_zc	Feline	4/15/21	B.1.1.7	North America / USA / Texas
21-012903-001v_zc	Feline	4/15/21	B.1.1.7	North America / USA / Texas
hCoV-19-USA-IL-UW-627/2020-EPI_zc	Human	6/26/20	B.1.139	North America / USA / Iowa / Jackson County
hCoV-19-USA-WI-GMF-00707/2020-EPI_zc	Human	4/6/20	B.1.308	North America / USA / Iowa / Allamakee County
hCoV-19-USA-WI-GMF-00744/2020-EPI_zc	Human	4/3/20	B.1.308	North America / USA / Iowa / Winneshiek County
hCoV-19-USA-WI-GMF-00857/2020-EPI_zc	Human	4/9/20	B.1	North America / USA / Iowa / Allamakee County
hCoV-19-USA-WI-GMF-00921/2020-EPI_zc	Human	4/3/20	B.1.308	North America / USA / Iowa / Allamakee County
hCoV-19-USA-WI-GMF-00928/2020-EPI_zc	Human	4/6/20	B.1.308	North America / USA / Iowa / Allamakee County
hCoV-19-USA-WI-GMF-01047/2020-EPI_zc	Human	4/14/20	B.1.308	North America / USA / Iowa / Allamakee County
hCoV-19-USA-WI-GMF-01158/2020-EPI_zc	Human	4/16/20	B.1.308	North America / USA / Iowa / Allamakee County
hCoV-19-USA-WI-GMF-01159/2020-EPI_zc	Human	4/16/20	B.1.308	North America / USA / Iowa / Allamakee County
hCoV-19-USA-WI-GMF-01316/2020-EPI_zc	Human	4/6/20	B.1.308	North America / USA / Iowa / Winneshiek County
hCoV-19-USA-WI-GMF-01455/2020-EPI_zc	Human	4/21/20	B.1.308	North America / USA / Iowa / Winneshiek County
hCoV-19-USA-WI-GMF-01534/2020-EPI_zc	Human	4/22/20	B.1.308	North America / USA / Iowa / Allamakee County
hCoV-19-USA-WI-GMF-01535/2020-EPI_zc	Human	4/21/20	B.1.308	North America / USA / Iowa / Allamakee County
hCoV-19-USA-WI-GMF-01551/2020-EPI_zc	Human	4/22/20	B.1.308	North America / USA / Iowa / Allamakee County
hCoV-19-USA-WI-GMF-01591/2020-EPI_zc	Human	4/23/20	B.1.308	North America / USA / Iowa / Allamakee County
hCoV-19-USA-WI-GMF-01828/2020-EPI_zc	Human	4/26/20	B.1.308	North America / USA / Iowa / Allamakee County
hCoV-19-USA-WI-GMF-01901/2020-EPI_zc	Human	4/27/20	B.1.308	North America / USA / Iowa / Allamakee County
hCoV-19-USA-WI-GMF-02381/2020-EPI_zc	Human	5/4/20	B.1	North America / USA / Iowa / Fayette County
hCoV-19-USA-WI-UW-1029/2020-EPI_zc	Human	unknown	unknown	unknown
hCoV-19-USA-WI-UW-1246/2020-EPI_zc	Human	unknown	unknown	unknown
hCoV-19-USA-WI-UW-1250/2020-EPI_zc	Human	unknown	unknown	unknown
hCoV-19-USA-WI-UW-1269/2020-EPI_zc	Human	unknown	unknown	unknown
hCoV-19-USA-WI-UW-1299/2020-EPI_zc	Human	unknown	unknown	unknown
hCoV-19-USA-WI-UW-1505/2020-EPI_zc	Human	unknown	unknown	unknown
hCoV-19-USA-WI-UW-1614/2020-EPI_zc	Human	unknown	unknown	unknown
hCoV-19-USA-WI-UW-2090/2020-EPI_zc	Human	unknown	unknown	unknown
hCoV-19-USA-WI-UW-3041/2021-EPI_zc	Human	unknown	unknown	unknown

Tree node ID	Host	Collection date	Lineage	Location
hCoV-19-USA-WI-UW-3100/2021-EPI_zc	Human	unknown	unknown	unknown
hCoV-19-USA-WI-UW-3348/2021-EPI_zc	Human	unknown	unknown	unknown
hCoV-19-USA-WI-WSLH-210137/2021-EPI_zc	Human	unknown	unknown	unknown
hCoV-19/USA/IA-10859/2020-EPI_zc	Human	5/4/20	B.1	North America / USA / Iowa
hCoV-19/USA/IA-9200028611/2020-EPI_zc	Human	8/14/20	B.1.2	North America / USA / Iowa
hCoV-19/USA/IA-9200056027/2020-EPI_zc	Human	9/16/20	B.1.2	North America / USA / Iowa
hCoV-19/USA/IA-9200064935/2020-EPI_zc	Human	9/24/20	B.1.240	North America / USA / Iowa
hCoV-19/USA/IA-9200064941/2020-EPI_zc	Human	9/24/20	B.1.240	North America / USA / Iowa
hCoV-19/USA/IA-9200086442/2020-EPI_zc	Human	10/13/20	B.1.587	North America / USA / Iowa
hCoV-19/USA/IA-9200130213/2020-EPI_zc	Human	11/23/20	B.1.595	North America / USA / Iowa
hCoV-19/USA/IA-9200156533/2020-EPI_zc	Human	12/23/20	B.1.2	North America / USA / Iowa
hCoV-19/USA/IA-9210074255/2020-EPI_zc	Human	12/9/20	B.1.565	North America / USA / Iowa
hCoV-19/USA/IA-9210074329/2020-EPI_zc	Human	10/28/20	B.1.243	North America / USA / Iowa
hCoV-19/USA/IA-9210074347/2020-EPI_zc	Human	10/28/20	B.1.240	North America / USA / Iowa
hCoV-19/USA/IA-9210074375/2020-EPI_zc	Human	9/28/20	B.1.2	North America / USA / Iowa
hCoV-19/USA/IA-9210074559/2020-EPI_zc	Human	9/8/20	B.1.2	North America / USA / Iowa
hCoV-19/USA/IA-9210074567/2020-EPI_zc	Human	10/16/20	B.1.565	North America / USA / Iowa
hCoV-19/USA/IA-CDC-2-3714295/2020-EPI_zc	Human	11/29/20	B.1.2	North America / USA / Iowa
hCoV-19/USA/IA-CDC-2-3714359/2020-EPI_zc	Human	11/28/20	B.1.311	North America / USA / Iowa
hCoV-19/USA/IA-CDC-2-3714367/2020-EPI_zc	Human	11/28/20	B.1.2	North America / USA / Iowa
hCoV-19/USA/IA-CDC-2-3714371/2020-EPI_zc	Human	11/29/20	B.1.2	North America / USA / Iowa
hCoV-19/USA/IA-CDC-2-3714376/2020-EPI_zc	Human	11/28/20	B.1.2	North America / USA / Iowa
hCoV-19/USA/IA-CDC-2-3714396/2020-EPI_zc	Human	11/29/20	B.1.2	North America / USA / Iowa
hCoV-19/USA/IA-CDC-2-3714458/2020-EPI_zc	Human	11/28/20	B.1.2	North America / USA / Iowa
hCoV-19/USA/IA-CDC-2-3714477/2020-EPI_zc	Human	11/27/20	B.1.2	North America / USA / Iowa
hCoV-19/USA/IA-CDC-2-3769189/2020-EPI_zc	Human	12/25/20	B.1.2	North America / USA / Iowa
hCoV-19/USA/IA-CDC-2-3769260/2020-EPI_zc	Human	12/24/20	B.1.565	North America / USA / Iowa
hCoV-19/USA/IA-CDC-2-3769271/2020-EPI_zc	Human	12/25/20	B.1.311	North America / USA / Iowa
hCoV-19/USA/IA-CDC-2-3769291/2020-EPI_zc	Human	12/26/20	B.1.396	North America / USA / Iowa
hCoV-19/USA/IA-CDC-2-3769295/2020-EPI_zc	Human	12/26/20	B.1.565	North America / USA / Iowa
hCoV-19/USA/IA-CDC-2-3845832/2021-EPI_zc	Human	1/27/21	B.1.2	North America / USA / Iowa
hCoV-19/USA/IA-CDC-2-3845834/2021-EPI_zc	Human	1/27/21	B.1.2	North America / USA / Iowa
hCoV-19/USA/IA-CDC-2-3845835/2021-EPI_zc	Human	1/27/21	B.1.2	North America / USA / Iowa
hCoV-19/USA/IA-CDC-2-3845912/2021-EPI_zc	Human	1/28/21	B.1.234	North America / USA / Iowa
hCoV-19/USA/IA-CDC-2-3845917/2021-EPI_zc	Human	1/28/21	B.1.2	North America / USA / Iowa
hCoV-19/USA/IA-CDC-2-3845921/2021-EPI_zc	Human	1/26/21	B.1.2	North America / USA / Iowa
hCoV-19/USA/IA-CDC-2-3845922/2021-EPI_zc	Human	1/27/21	B.1.2	North America / USA / Iowa
hCoV-19/USA/IA-CDC-2-3845930/2021-EPI_zc	Human	1/26/21	B.1.2	North America / USA / Iowa
hCoV-19/USA/IA-CDC-2-3846010/2021-EPI_zc	Human	1/27/21	B.1.2	North America / USA / Iowa

Tree node ID	Host	Collection date	Lineage	Location
hCoV-19/USA/IA-CDC-2-3881101/2021-EPI_zc	Human	2/1/21	B.1.234	North America / USA / Iowa
hCoV-19/USA/IA-CDC-2-3881114/2021-EPI_zc	Human	2/1/21	B.1.427	North America / USA / Iowa
hCoV-19/USA/IA-CDC-2-3881213/2021-EPI_zc	Human	2/2/21	B.1.311	North America / USA / Iowa
hCoV-19/USA/IA-CDC-2-3881220/2021-EPI_zc	Human	2/2/21	B.1.1.519	North America / USA / Iowa
hCoV-19/USA/IA-CDC-2-3972992/2021-EPI_zc	Human	2/17/21	B.1.2	North America / USA / Iowa
hCoV-19/USA/IA-CDC-2-3972996/2021-EPI_zc	Human	2/18/21	B.1.2	North America / USA / Iowa
hCoV-19/USA/IA-CDC-2-3973074/2021-EPI_zc	Human	2/19/21	B.1.2	North America / USA / Iowa
hCoV-19/USA/IA-CDC-2-3973077/2021-EPI_zc	Human	2/17/21	B.1.2	North America / USA / Iowa
hCoV-19/USA/IA-CDC-2-3973078/2021-EPI_zc	Human	2/19/21	B.1.2	North America / USA / Iowa
hCoV-19/USA/IA-CDC-2-3973092/2021-EPI_zc	Human	2/18/21	B.1.429	North America / USA / Iowa
hCoV-19/USA/IA-CDC-2-3979370/2021-EPI_zc	Human	2/24/21	B.1.311	North America / USA / Iowa
hCoV-19/USA/IA-CDC-2-3979374/2021-EPI_zc	Human	2/23/21	B.1	North America / USA / Iowa
hCoV-19/USA/IA-CDC-2-3979452/2021-EPI_zc	Human	2/23/21	B.1.429	North America / USA / Iowa
hCoV-19/USA/IA-CDC-2-3979455/2021-EPI_zc	Human	2/24/21	B.1.2	North America / USA / Iowa
hCoV-19/USA/IA-CDC-2-3979456/2021-EPI_zc	Human	2/23/21	B.1.2	North America / USA / Iowa
hCoV-19/USA/IA-CDC-2-3979461/2021-EPI_zc	Human	2/24/21	B.1.2	North America / USA / Iowa
hCoV-19/USA/IA-CDC-2-3979470/2021-EPI_zc	Human	2/22/21	B.1.575	North America / USA / Iowa
hCoV-19/USA/IA-CDC-2-3979472/2021-EPI_zc	Human	2/25/21	B.1.2	North America / USA / Iowa
hCoV-19/USA/IA-CDC-2-3979499/2021-EPI_zc	Human	2/25/21	B.1.2	North America / USA / Iowa
hCoV-19/USA/IA-CDC-2-4014855/2021-EPI_zc	Human	2/27/21	B.1.2	North America / USA / Iowa
hCoV-19/USA/IA-CDC-2-4044425/2020-EPI_zc	Human	12/14/20	B.1.2	North America / USA / Iowa
hCoV-19/USA/IA-CDC-2-4044433/2020-EPI_zc	Human	12/14/20	B.1.565	North America / USA / Iowa
hCoV-19/USA/IA-CDC-2-4044443/2020-EPI_zc	Human	12/18/20	B.1.234	North America / USA / Iowa
hCoV-19/USA/IA-CDC-2-4044501/2020-EPI_zc	Human	12/12/20	B.1.2	North America / USA / Iowa
hCoV-19/USA/IA-CDC-2-4044533/2020-EPI_zc	Human	12/14/20	B.1.2	North America / USA / Iowa
hCoV-19/USA/IA-CDC-2-4069001/2021-EPI_zc	Human	1/14/21	B.1.234	North America / USA / Iowa
hCoV-19/USA/IA-CDC-2-4069002/2021-EPI_zc	Human	1/14/21	B.1.1.316	North America / USA / Iowa
hCoV-19/USA/IA-CDC-2-4069008/2021-EPI_zc	Human	1/15/21	B.1.234	North America / USA / Iowa
hCoV-19/USA/IA-CDC-2-4069009/2021-EPI_zc	Human	1/14/21	B.1.2	North America / USA / Iowa
hCoV-19/USA/IA-CDC-2-4069022/2021-EPI_zc	Human	1/16/21	B.1.2	North America / USA / Iowa
hCoV-19/USA/IA-CDC-2-4069023/2021-EPI_zc	Human	1/14/21	B.1.565	North America / USA / Iowa
hCoV-19/USA/IA-CDC-2-4069028/2021-EPI_zc	Human	1/14/21	B.1.1.316	North America / USA / Iowa
hCoV-19/USA/IA-CDC-2-4069031/2021-EPI_zc	Human	1/15/21	B.1.2	North America / USA / Iowa
hCoV-19/USA/IA-CDC-2-4069049/2021-EPI_zc	Human	1/15/21	B.1.2	North America / USA / Iowa
hCoV-19/USA/IA-CDC-2-4069108/2021-EPI_zc	Human	1/15/21	B.1.2	North America / USA / Iowa
hCoV-19/USA/IA-CDC-2-4069110/2021-EPI_zc	Human	1/14/21	B.1.2	North America / USA / Iowa
hCoV-19/USA/IA-CDC-2-4069120/2021-EPI_zc	Human	1/15/21	B.1.2	North America / USA / Iowa
hCoV-19/USA/IA-CDC-2-4069121/2021-EPI_zc	Human	1/15/21	B.1.2	North America / USA / Iowa
hCoV-19/USA/IA-CDC-2-4069126/2021-EPI_zc	Human	1/15/21	B.1.311	North America / USA / Iowa

Tree node ID	Host	Collection date	Lineage	Location
hCoV-19/USA/IA-CDC-2-4634493/2021-EPI_zc	Human	2/9/21	B.1.1.351	North America / USA / Iowa
hCoV-19/USA/IA-CDC-2-4634554/2021-EPI_zc	Human	2/11/21	B.1.2	North America / USA / Iowa
hCoV-19/USA/IA-CDC-2-4634565/2021-EPI_zc	Human	2/8/21	B.1.2	North America / USA / Iowa
hCoV-19/USA/IA-CDC-2-4634583/2021-EPI_zc	Human	2/10/21	B.1.2	North America / USA / Iowa
hCoV-19/USA/IA-CDC-2-4634589/2021-EPI_zc	Human	2/10/21	B.1.427	North America / USA / Iowa
hCoV-19/USA/IA-CDC-2-4634591/2021-EPI_zc	Human	2/10/21	B.1.1.519	North America / USA / Iowa
hCoV-19/USA/IA-CDC-2-4634651/2021-EPI_zc	Human	2/10/21	B.1.2	North America / USA / Iowa
hCoV-19/USA/IA-CDC-2-4634671/2021-EPI_zc	Human	2/8/21	B.1.2	North America / USA / Iowa
hCoV-19/USA/IA-CDC-ASC210000174/2021-EPI_zc	Human	2/28/21	B.1.2	North America / USA / Iowa
hCoV-19/USA/IA-CDC-ASC210000175/2021-EPI_zc	Human	2/28/21	B.1.311	North America / USA / Iowa
hCoV-19/USA/IA-CDC-ASC210000176/2021-EPI_zc	Human	2/28/21	B.1.2	North America / USA / Iowa
hCoV-19/USA/IA-CDC-ASC210000327/2021-EPI_zc	Human	2/28/21	B.1.1.519	North America / USA / Iowa
hCoV-19/USA/IA-CDC-LC00002729/2021-EPI_zc	Human	1/4/21	B.1.2	North America / USA / Iowa
hCoV-19/USA/IA-CDC-LC0000857/2020-EPI_zc	Human	12/23/20	B.1.2	North America / USA / Iowa
hCoV-19/USA/IA-CDC-LC0002727/2021-EPI_zc	Human	1/4/21	B.1.2	North America / USA / Iowa
hCoV-19/USA/IA-CDC-LC0002729/2021-EPI_zc	Human	1/4/21	B.1.2	North America / USA / Iowa
hCoV-19/USA/IA-CDC-LC0002732/2021-EPI_zc	Human	1/4/21	B.1.2	North America / USA / Iowa
hCoV-19/USA/IA-CDC-LC0003220/2021-EPI_zc	Human	1/10/21	B.1	North America / USA / Iowa
hCoV-19/USA/IA-CDC-LC0004145/2021-EPI_zc	Human	1/2/21	B.1.2	North America / USA / Iowa
hCoV-19/USA/IA-CDC-LC0004752/2020-EPI_zc	Human	12/31/20	B.1.2	North America / USA / Iowa
hCoV-19/USA/IA-CDC-LC0008648/2021-EPI_zc	Human	1/20/21	B.1.2	North America / USA / Iowa
hCoV-19/USA/IA-CDC-LC0008664/2021-EPI_zc	Human	1/20/21	B.1.2	North America / USA / Iowa
hCoV-19/USA/IA-CDC-LC0008665/2021-EPI_zc	Human	1/20/21	B.1.427	North America / USA / Iowa
hCoV-19/USA/IA-CDC-LC0008670/2021-EPI_zc	Human	1/19/21	B.1.2	North America / USA / Iowa
hCoV-19/USA/IA-CDC-LC0009085/2021-EPI_zc	Human	1/21/21	B.1.2	North America / USA / Iowa
hCoV-19/USA/IA-CDC-LC0009089/2021-EPI_zc	Human	1/21/21	B.1.427	North America / USA / Iowa
hCoV-19/USA/IA-CDC-LC0011536/2021-EPI_zc	Human	1/28/21	B.1.596	North America / USA / Iowa
hCoV-19/USA/IA-CDC-LC0011643/2021-EPI_zc	Human	1/29/21	B.1.1.416	North America / USA / Iowa
hCoV-19/USA/IA-CDC-LC0012002/2021-EPI_zc	Human	1/29/21	B.1.2	North America / USA / Iowa
hCoV-19/USA/IA-CDC-LC0012003/2021-EPI_zc	Human	1/29/21	B.1.234	North America / USA / Iowa
hCoV-19/USA/IA-CDC-LC0012014/2021-EPI_zc	Human	1/29/21	B.1.427	North America / USA / Iowa
hCoV-19/USA/IA-CDC-LC0012977/2021-EPI_zc	Human	2/1/21	B.1.2	North America / USA / Iowa
hCoV-19/USA/IA-CDC-LC0012978/2021-EPI_zc	Human	2/1/21	B.1.2	North America / USA / Iowa
hCoV-19/USA/IA-CDC-LC0012979/2021-EPI_zc	Human	2/1/21	B.1.2	North America / USA / Iowa
hCoV-19/USA/IA-CDC-LC0020323/2021-EPI_zc	Human	2/22/21	B.1.2	North America / USA / Iowa
hCoV-19/USA/IA-CDC-QDX21533090/2021-EPI_zc	Human	1/27/21	B.1.139	North America / USA / Iowa
hCoV-19/USA/IA-CDC-QDX22214251/2021-EPI_zc	Human	2/17/21	B.1.234	North America / USA / Iowa
hCoV-19/USA/IA-CDC-QDX22315846/2021-EPI_zc	Human	2/22/21	B.1.429	North America / USA / Iowa
hCoV-19/USA/IA-CDC-STM-0000013-D03/2021-EPI_zc	Human	1/14/21	B.1.2	North America / USA / Iowa

Tree node ID	Host	Collection date	Lineage	Location
hCoV-19/USA/IA-CDC-STM-000002415/2021-EPI_zc	Human	1/14/21	B.1.1.519	North America / USA / Iowa
hCoV-19/USA/IA-CDC-STM-000005050/2021-EPI_zc	Human	1/22/21	B.1.2	North America / USA / Iowa
hCoV-19/USA/IA-CDC-STM-000005985/2021-EPI_zc	Human	1/28/21	B.1.2	North America / USA / Iowa
hCoV-19/USA/IA-CDC-STM-000023171/2021-EPI_zc	Human	2/24/21	B.1.1.7	North America / USA / Iowa
hCoV-19/USA/IA-CDC-STM-A019/2021-EPI_zc	Human	1/2/21	B.1.2	North America / USA / Iowa
hCoV-19/USA/IA-CDC-STM-A100135/2021-EPI_zc	Human	1/4/21	B.1.2	North America / USA / Iowa
hCoV-19/USA/IA-CDC-STM-A100382/2021-EPI_zc	Human	1/6/21	B.1.2	North America / USA / Iowa
hCoV-19/USA/IA-GMF-02699/2020-EPI_zc	Human	5/8/20	B.1.308	North America / USA / Iowa / Allamakee County
hCoV-19/USA/IA-GMF-08606/2020-EPI_zc	Human	6/29/20	B.1.401	North America / USA / Iowa / Allamakee County
hCoV-19/USA/IA-GMF-08792/2020-EPI_zc	Human	7/1/20	B.1.401	North America / USA / Iowa / Fayette County
hCoV-19/USA/IA-GMF-08809/2020-EPI_zc	Human	7/1/20	B.1.401	North America / USA / Iowa / Fayette County
hCoV-19/USA/IA-GMF-09513/2020-EPI_zc	Human	7/6/20	B.1.401	North America / USA / Iowa / Fayette County
hCoV-19/USA/IA-GMF-09911/2020-EPI_zc	Human	7/8/20	B.1.413	North America / USA / Iowa / Fayette County
hCoV-19/USA/IA-GMF-10147/2020-EPI_zc	Human	7/10/20	B.1.413	North America / USA / Iowa / Fayette County
hCoV-19/USA/IA-GMF-11334/2020-EPI_zc	Human	7/18/20	B.1.565	North America / USA / Iowa / Winneshiek County
hCoV-19/USA/IA-GMF-12677/2020-EPI_zc	Human	7/27/20	B.1.565	North America / USA / Iowa / Winneshiek County
hCoV-19/USA/IA-GMF-13403/2020-EPI_zc	Human	8/1/20	B.1.582	North America / USA / Iowa / Clayton County
hCoV-19/USA/IA-GMF-13859/2020-EPI_zc	Human	8/4/20	B.1.565	North America / USA / Iowa / Allamakee County
hCoV-19/USA/IA-GMF-14006/2020-EPI_zc	Human	8/5/20	B.1.369	North America / USA / Iowa / Allamakee County
hCoV-19/USA/IA-GMF-15466/2020-EPI_zc	Human	8/14/20	B.1.110.3	North America / USA / Iowa / Fayette County
hCoV-19/USA/IA-GMF-15767/2020-EPI_zc	Human	8/17/20	B.1.582	North America / USA / Iowa / Fayette County
hCoV-19/USA/IA-GMF-16252/2020-EPI_zc	Human	8/20/20	B.1.110.3	North America / USA / Iowa / Winneshiek County
hCoV-19/USA/IA-GMF-16267/2020-EPI_zc	Human	8/19/20	B.1.110.3	North America / USA / Iowa / Fayette County
hCoV-19/USA/IA-GMF-16489/2020-EPI_zc	Human	8/21/20	B.1.110.3	North America / USA / Iowa / Fayette County
hCoV-19/USA/IA-GMF-17036/2020-EPI_zc	Human	8/24/20	B.1.240	North America / USA / Iowa / Allamakee County
hCoV-19/USA/IA-GMF-18219/2020-EPI_zc	Human	8/31/20	B.1.565	North America / USA / Iowa / Fayette County
hCoV-19/USA/IA-GMF-18345/2020-EPI_zc	Human	9/1/20	B.1.565	North America / USA / Iowa / Fayette County
hCoV-19/USA/IA-GMF-18509/2020-EPI_zc	Human	9/2/20	B.1.110.3	North America / USA / Iowa / Winneshiek County
hCoV-19/USA/IA-GMF-18813/2020-EPI_zc	Human	9/5/20	B.1.565	North America / USA / Iowa / Howard County
hCoV-19/USA/IA-GMF-18906/2020-EPI_zc	Human	9/5/20	B.1.587	North America / USA / Iowa / Clayton County
hCoV-19/USA/IA-GMF-18913/2020-EPI_zc	Human	9/4/20	B.1.1.222	North America / USA / Iowa / Fayette County
hCoV-19/USA/IA-GMF-19239/2020-EPI_zc	Human	9/8/20	B.1.2	North America / USA / Iowa / Johnson County
hCoV-19/USA/IA-GMF-21220/2020-EPI_zc	Human	9/19/20	B.1.1.464	North America / USA / Iowa / La Crosse County
hCoV-19/USA/IA-GMF-21344/2020-EPI_zc	Human	9/20/20	B.1.582	North America / USA / Iowa / Winneshiek County
hCoV-19/USA/IA-GMF-23570/2020-EPI_zc	Human	10/2/20	B.1.2	North America / USA / Iowa / Winneshiek County
hCoV-19/USA/IA-GMF-23647/2020-EPI_zc	Human	10/2/20	B.1.565	North America / USA / Iowa / Allamakee County
hCoV-19/USA/IA-GMF-24556/2020-EPI_zc	Human	10/7/20	B.1.565	North America / USA / Iowa / Fayette County
hCoV-19/USA/IA-GMF-25157/2020-EPI_zc	Human	10/11/20	B.1.2	North America / USA / Iowa / Allamakee County
hCoV-19/USA/IA-GMF-25923/2020-EPI_zc	Human	10/15/20	B.1.565	North America / USA / Iowa / Clayton County

Tree node ID	Host	Collection date	Lineage	Location
hCoV-19/USA/IA-GMF-25958/2020-EPI_zc	Human	10/15/20	B.1.2	North America / USA / Iowa / Allamakee County
hCoV-19/USA/IA-GMF-26895/2020-EPI_zc	Human	10/20/20	B.1.240	North America / USA / Iowa / Allamakee County
hCoV-19/USA/IA-GMF-27699/2020-EPI_zc	Human	10/25/20	B.1.2	North America / USA / Iowa / Allamakee County
hCoV-19/USA/IA-GMF-28410/2020-EPI_zc	Human	10/28/20	B.1.2	North America / USA / Iowa / Allamakee County
hCoV-19/USA/IA-GMF-28426/2020-EPI_zc	Human	10/28/20	B.1.2	North America / USA / Iowa / Allamakee County
hCoV-19/USA/IA-GMF-31330/2020-EPI_zc	Human	11/4/20	B.1.2	North America / USA / Iowa / Allamakee County
hCoV-19/USA/IA-GMF-31421/2020-EPI_zc	Human	11/4/20	B.1.2	North America / USA / Iowa / Allamakee County
hCoV-19/USA/IA-GMF-31654/2020-EPI_zc	Human	11/5/20	B.1.2	North America / USA / Iowa / Allamakee County
hCoV-19/USA/IA-GMF-32028/2020-EPI_zc	Human	11/7/20	B.1	North America / USA / Iowa / Winneshiek County
hCoV-19/USA/IA-GMF-32164/2020-EPI_zc	Human	11/7/20	B.1.2	North America / USA / Iowa / Allamakee County
hCoV-19/USA/IA-GMF-32395/2020-EPI_zc	Human	11/9/20	B.1.2	North America / USA / Iowa / Winneshiek County
hCoV-19/USA/IA-GMF-32590/2020-EPI_zc	Human	11/9/20	B.1.139	North America / USA / Iowa / Allamakee County
hCoV-19/USA/IA-GMF-32700/2020-EPI_zc	Human	11/9/20	B.1.396	North America / USA / Iowa / Winneshiek County
hCoV-19/USA/IA-GMF-32907/2020-EPI_zc	Human	11/10/20	B.1.2	North America / USA / Iowa / Allamakee County
hCoV-19/USA/IA-GMF-33129/2020-EPI_zc	Human	11/11/20	B.1	North America / USA / Iowa / Clayton County
hCoV-19/USA/IA-GMF-33366/2020-EPI_zc	Human	11/11/20	B.1.241	North America / USA / Iowa / Dubuque County
hCoV-19/USA/IA-GMF-33487/2020-EPI_zc	Human	11/11/20	B.1.110.3	North America / USA / Iowa / Clayton County
hCoV-19/USA/IA-GMF-34475/2020-EPI_zc	Human	11/15/20	B.1.565	North America / USA / Iowa / Chickasaw County
hCoV-19/USA/IA-GMF-34972/2020-EPI_zc	Human	11/16/20	B.1.2	North America / USA / Iowa / Fayette County
hCoV-19/USA/IA-GMF-36354/2020-EPI_zc	Human	11/22/20	B.1.2	North America / USA / Iowa / Allamakee County
hCoV-19/USA/IA-GMF-36674/2020-EPI_zc	Human	11/24/20	B.1.2	North America / USA / Iowa / Allamakee County
hCoV-19/USA/IA-GMF-37933/2020-EPI_zc	Human	11/30/20	B.1.564	North America / USA / Iowa / Howard County
hCoV-19/USA/IA-GMF-38015/2020-EPI_zc	Human	11/30/20	B.1.2	North America / USA / Iowa / Allamakee County
hCoV-19/USA/IA-GMF-38206/2020-EPI_zc	Human	12/1/20	B.1.2	North America / USA / Iowa / Allamakee County
hCoV-19/USA/IA-GMF-3912/2020-EPI_zc	Human	5/20/20	B.1	North America / USA / Iowa / Buchanan County
hCoV-19/USA/IA-GMF-41299/2020-EPI_zc	Human	12/11/20	B.1.596	North America / USA / Iowa / Winneshiek County
hCoV-19/USA/IA-GMF-41403/2020-EPI_zc	Human	12/12/20	B.1.110.3	North America / USA / Iowa / Winneshiek County
hCoV-19/USA/IA-GMF-43363/2020-EPI_zc	Human	12/19/20	B.1.2	North America / USA / Iowa / Winneshiek County
hCoV-19/USA/IA-GMF-46261/2021-EPI_zc	Human	1/3/21	B.1.2	North America / USA / Iowa / Allamakee County
hCoV-19/USA/IA-GMF-46406/2021-EPI_zc	Human	1/3/21	R.1	North America / USA / Iowa / Fayette County
hCoV-19/USA/IA-GMF-47194/2021-EPI_zc	Human	1/5/21	B.1.2	North America / USA / Iowa / Allamakee County
hCoV-19/USA/IA-GMF-47250/2021-EPI_zc	Human	1/5/21	R.1	North America / USA / Iowa / Winneshiek County
hCoV-19/USA/IA-GMF-48948/2021-EPI_zc	Human	1/12/21	B.1.2	North America / USA / Iowa / Winneshiek County
hCoV-19/USA/IA-GMF-48951/2021-EPI_zc	Human	1/12/21	B.1.2	North America / USA / Iowa / Fayette County
hCoV-19/USA/IA-GMF-49259/2021-EPI_zc	Human	1/14/21	B.1.2	North America / USA / Iowa / Fayette County
hCoV-19/USA/IA-GMF-49459/2021-EPI_zc	Human	1/15/21	B.1.2	North America / USA / Iowa / Winneshiek County
hCoV-19/USA/IA-GMF-49508/2021-EPI_zc	Human	1/16/21	B.1.2	North America / USA / Iowa / Fayette County
hCoV-19/USA/IA-GMF-49510/2021-EPI_zc	Human	1/16/21	B.1.2	North America / USA / Iowa / Fayette County
hCoV-19/USA/IA-GMF-49586/2021-EPI_zc	Human	1/16/21	B.1.427	North America / USA / Iowa / Allamakee County

Tree node ID	Host	Collection date	Lineage	Location
hCoV-19/USA/IA-GMF-50144/2021-EPI_zc	Human	1/18/21	B.1.2	North America / USA / Iowa / Fayette County
hCoV-19/USA/IA-GMF-50146/2021-EPI_zc	Human	1/18/21	B.1.2	North America / USA / Iowa / Fayette County
hCoV-19/USA/IA-GMF-50340/2021-EPI_zc	Human	1/19/21	B.1.2	North America / USA / Iowa / Fayette County
hCoV-19/USA/IA-GMF-50854/2021-EPI_zc	Human	1/21/21	B.1.2	North America / USA / Iowa / Allamakee County
hCoV-19/USA/IA-GMF-52611/2021-EPI_zc	Human	1/29/21	B.1.2	North America / USA / Iowa / Winneshiek County
hCoV-19/USA/IA-GMF-54488/2021-EPI_zc	Human	2/15/21	B.1.427	North America / USA / Iowa / Winneshiek County
hCoV-19/USA/IA-GMF-54489/2021-EPI_zc	Human	2/15/21	B.1.2	North America / USA / Iowa / Fayette County
hCoV-19/USA/IA-GMF-54550/2021-EPI_zc	Human	2/16/21	B.1.2	North America / USA / Iowa / Winneshiek County
hCoV-19/USA/IA-GMF-55353/2021-EPI_zc	Human	2/23/21	B.1.2	North America / USA / Iowa / Allamakee County
hCoV-19/USA/IA-GMF-55463/2021-EPI_zc	Human	2/24/21	B.1.2	North America / USA / Iowa / Fayette County
hCoV-19/USA/IA-GMF-B00025/2021-EPI_zc	Human	2/14/21	B.1.2	North America / USA / Iowa / Fayette County
hCoV-19/USA/IA-GMF-B00028/2021-EPI_zc	Human	2/16/21	B.1.2	North America / USA / Iowa / Fayette County
hCoV-19/USA/IA-GMF-B00034/2021-EPI_zc	Human	2/18/21	B.1.2	North America / USA / Iowa / Winneshiek County
hCoV-19/USA/IA-GMF-B00035/2021-EPI_zc	Human	2/19/21	B.1.234	North America / USA / Iowa / Winneshiek County
hCoV-19/USA/IA-GMF-M00005/2020-EPI_zc	Human	5/26/20	B.1.308	North America / USA / Iowa / Allamakee County
hCoV-19/USA/IA-GMF/20217/2020-EPI_zc	Human	5/26/20	B.1.308	North America / USA / Iowa / Allamakee County
hCoV-19/USA/IA-Noblis-S369B17/2021-EPI_zc	Human	2/23/21	B.1.234	North America / USA / Iowa
hCoV-19/USA/IA-Noblis-S371B18/2021-EPI_zc	Human	2/23/21	B.1.234	North America / USA / Iowa
hCoV-19/USA/IA-Noblis-S51B02/2020-EPI_zc	Human	10/29/20	B.1.2	North America / USA / Iowa
hCoV-19/USA/IA-Noblis-S52B03/2020-EPI_zc	Human	10/29/20	B.1.2	North America / USA / Iowa
hCoV-19/USA/IA-Noblis-S59B12/2020-EPI_zc	Human	12/2/20	B.1.2	North America / USA / Iowa
hCoV-19/USA/IA-Noblis-S61B14/2020-EPI_zc	Human	12/2/20	B.1.2	North America / USA / Iowa
hCoV-19/USA/IA-Noblis-S62B15/2020-EPI_zc	Human	12/20/20	B.1.2	North America / USA / Iowa
hCoV-19/USA/IA-QDX-249/2020-EPI_zc	Human	4/29/20	B.1	North America / USA / Iowa
hCoV-19/USA/IA-QDX-4437/2020-EPI_zc	Human	11/7/20	B.1.2	North America / USA / Iowa
hCoV-19/USA/IA-SHL-1013770/2020-EPI_zc	Human	5/18/20	B.1.382	North America / USA / Iowa
hCoV-19/USA/IA-SHL-1079193/2020-EPI_zc	Human	6/29/20	B.1.564	North America / USA / Iowa
hCoV-19/USA/IA-SHL-1294465/2020-EPI_zc	Human	9/25/20	B.1.565	North America / USA / Iowa
hCoV-19/USA/IA-SHL-1369391/2020-EPI_zc	Human	10/26/20	B.1.369	North America / USA / Iowa
hCoV-19/USA/IA-SHL-1377598/2020-EPI_zc	Human	10/29/20	B.1.564	North America / USA / Iowa
hCoV-19/USA/IA-SHL-1384887/2020-EPI_zc	Human	11/3/20	B.1.565	North America / USA / Iowa
hCoV-19/USA/IA-SHL-1560811/2021-EPI_zc	Human	1/20/21	B.1.2	North America / USA / Iowa
hCoV-19/USA/IA-SHL-1560839/2021-EPI_zc	Human	1/23/21	B.1.2	North America / USA / Iowa
hCoV-19/USA/IA-SHL-1560855/2021-EPI_zc	Human	1/24/21	B.1.2	North America / USA / Iowa
hCoV-19/USA/IA-SHL-1561390/2021-EPI_zc	Human	1/25/21	B.1.234	North America / USA / Iowa
hCoV-19/USA/IA-SHL-1561772/2021-EPI_zc	Human	1/25/21	B.1.2	North America / USA / Iowa / Johnson
hCoV-19/USA/IA-SHL-1561778/2021-EPI_zc	Human	1/23/21	B.1.2	North America / USA / Iowa
hCoV-19/USA/IA-SHL-1561786/2021-EPI_zc	Human	1/23/21	B.1.2	North America / USA / Iowa
hCoV-19/USA/IA-SHL-1565144/2021-EPI_zc	Human	1/17/21	B.1.1.7	North America / USA / Iowa / Johnson

Tree node ID	Host	Collection date	Lineage	Location
hCoV-19/USA/IA-SHL-1565147/2021-EPI_zc	Human	1/6/21	B.1.1.7	North America / USA / Iowa / Johnson
hCoV-19/USA/IA-SHL-1565149/2021-EPI_zc	Human	1/21/21	B.1.1.7	North America / USA / Iowa / Bremer
hCoV-19/USA/IA-SHL-1570505/2021-EPI_zc	Human	1/28/21	B.1.2	North America / USA / Iowa / Black Hawk
hCoV-19/USA/IA-SHL-1570506/2021-EPI_zc	Human	1/28/21	B.1.234	North America / USA / Iowa / Buchanan
hCoV-19/USA/IA-SHL-1570723/2021-EPI_zc	Human	1/31/21	B.1.1.7	North America / USA / Iowa / Winneshiek
hCoV-19/USA/IA-SHL-1573391/2021-EPI_zc	Human	1/5/21	B.1.1.7	North America / USA / Iowa / Unknown
hCoV-19/USA/IA-SHL-1574118/2021-EPI_zc	Human	2/1/21	B.1.2	North America / USA / Iowa / Dubuque
hCoV-19/USA/IA-SHL-1574119/2021-EPI_zc	Human	2/1/21	B.1.2	North America / USA / Iowa / Dubuque
hCoV-19/USA/IA-SHL-1574132/2021-EPI_zc	Human	2/1/21	B.1.2	North America / USA / Iowa / Dubuque
hCoV-19/USA/IA-SHL-1574134/2021-EPI_zc	Human	2/1/21	B.1.2	North America / USA / Iowa / Dubuque
hCoV-19/USA/IA-SHL-1574135/2021-EPI_zc	Human	2/1/21	B.1.2	North America / USA / Iowa / Dubuque
hCoV-19/USA/IA-SHL-1574323/2021-EPI_zc	Human	2/1/21	B.1.2	North America / USA / Iowa / Dubuque
hCoV-19/USA/IA-SHL-1576764/2021-EPI_zc	Human	1/27/21	B.1.1.7	North America / USA / Iowa / Polk
hCoV-19/USA/IA-SHL-1602606/2021-EPI_zc	Human	2/25/21	B.1.2	North America / USA / Iowa
hCoV-19/USA/IA-SHL-1602666/2021-EPI_zc	Human	2/25/21	B.1.2	North America / USA / Iowa
hCoV-19/USA/IA-SHL-1780926/2021-EPI_zc	Human	2/8/21	AY.39	North America / USA / Iowa
hCoV-19/USA/IA-UIHC-MO01/2020-EPI_zc	Human	6/8/20	B.1	North America / USA / Iowa / Johnson County
hCoV-19/USA/IA-UIHC-MO02/2020-EPI_zc	Human	6/8/20	B.1	North America / USA / Iowa / Johnson County
hCoV-19/USA/IA-UIHC-MO03/2020-EPI_zc	Human	6/9/20	B.1	North America / USA / Iowa / Johnson County
hCoV-19/USA/IA-UIHC-MO04/2020-EPI_zc	Human	6/9/20	B.1	North America / USA / Iowa / Johnson County
hCoV-19/USA/IA-UIHC-MO05/2020-EPI_zc	Human	6/10/20	B.1	North America / USA / Iowa / Johnson County
hCoV-19/USA/IA-UIHC-MO06/2020-EPI_zc	Human	6/11/20	B.1	North America / USA / Iowa / Johnson County
hCoV-19/USA/IA-UIHC-MO07/2020-EPI_zc	Human	6/11/20	B.1	North America / USA / Iowa / Johnson County
hCoV-19/USA/IA-UIHC-MO08/2020-EPI_zc	Human	6/11/20	B.1	North America / USA / Iowa / Johnson County
hCoV-19/USA/IA-UIHC-MO09/2020-EPI_zc	Human	6/12/20	B.1	North America / USA / Iowa / Johnson County
hCoV-19/USA/IA-UIHC-MO10/2020-EPI_zc	Human	6/12/20	B.1	North America / USA / Iowa / Johnson County
hCoV-19/USA/IA-UIHC-MO11/2020-EPI_zc	Human	6/14/20	B.1	North America / USA / Iowa / Johnson County
hCoV-19/USA/IA-UIHC-MO12/2020-EPI_zc	Human	6/15/20	B.1	North America / USA / Iowa / Johnson County
hCoV-19/USA/IA-UIHC-MO13/2020-EPI_zc	Human	6/16/20	B.1	North America / USA / Iowa / Johnson County
hCoV-19/USA/IA-UIHC-MO14/2020-EPI_zc	Human	6/16/20	B.1	North America / USA / Iowa / Johnson County
hCoV-19/USA/IA-UIHC-MO15/2020-EPI_zc	Human	6/16/20	B.1	North America / USA / Iowa / Johnson County
hCoV-19/USA/IA-UIHC-MO16/2020-EPI_zc	Human	6/17/20	B.1	North America / USA / Iowa / Johnson County
hCoV-19/USA/IA-UIHC-MO17/2020-EPI_zc	Human	6/18/20	B.1	North America / USA / Iowa / Johnson County
hCoV-19/USA/IA-UIHC-MO18/2020-EPI_zc	Human	9/2/20	B.1.565	North America / USA / Iowa / Johnson County
hCoV-19/USA/IA-UIHC-MO19/2020-EPI_zc	Human	9/3/20	B.1.565	North America / USA / Iowa / Johnson County
hCoV-19/USA/IA-UIHC-MO20/2020-EPI_zc	Human	9/4/20	B.1.582	North America / USA / Iowa / Johnson County
hCoV-19/USA/IA-UIHC-MO21/2020-EPI_zc	Human	9/5/20	B.1.565	North America / USA / Iowa / Johnson County
hCoV-19/USA/IA-UIHC-MO22/2020-EPI_zc	Human	9/5/20	B.1.565	North America / USA / Iowa / Johnson County
hCoV-19/USA/IA-UIHC-MO23/2020-EPI_zc	Human	9/6/20	B.1.565	North America / USA / Iowa / Johnson County

Tree node ID	Host	Collection date	Lineage	Location
hCoV-19/USA/IA-UIHC-MO24/2020-EPI_zc	Human	9/9/20	B.1.565	North America / USA / Iowa / Johnson County
hCoV-19/USA/IA-UIHC-MO25/2020-EPI_zc	Human	9/9/20	B.1.565	North America / USA / Iowa / Johnson County
hCoV-19/USA/IA-UIHC-MO26/2020-EPI_zc	Human	9/10/20	B.1.565	North America / USA / Iowa / Johnson County
hCoV-19/USA/IA-UIHC-MO27/2020-EPI_zc	Human	9/10/20	B.1.565	North America / USA / Iowa / Johnson County
hCoV-19/USA/IA-UIHC-MO28/2020-EPI_zc	Human	9/10/20	B.1.565	North America / USA / Iowa / Johnson County
hCoV-19/USA/IA-UIHC-MO29/2020-EPI_zc	Human	5/11/20	B.1	North America / USA / Iowa / Johnson County
hCoV-19/USA/IA-UIHC-MO30/2020-EPI_zc	Human	7/22/20	B.1.2	North America / USA / Iowa / Johnson County
hCoV-19/USA/IA-UIHC-MO31/2020-EPI_zc	Human	11/23/20	B.1.2	North America / USA / Iowa / Johnson County
hCoV-19/USA/IA-UIHC-MO32/2020-EPI_zc	Human	11/25/20	B.1.2	North America / USA / Iowa / Johnson County
hCoV-19/USA/IA-UW-121/2020-EPI_zc	Human	4/11/20	B.1	North America / USA / Iowa / Dubuque County
hCoV-19/USA/IA-UW-2458/2020-EPI_zc	Human	12/22/20	B.1.2	North America / USA / Iowa / Fayette County
21-010357-002s_lion_04-04-21_PA_fc	Lion	4/4/21	B.1.1.7	North America / USA / Pennsylvania
20-022930-002_mink2_8-10-20_UT_F2_Index	Mink	8/10/20	B.1	North America / USA / Utah
20-022930-005_mink2_8-10-20_UT_F3_Index	Mink	8/10/20	B.1	North America / USA / Utah
20-022930-009v_mink2_UT_F3_Index	Mink	8/10/20	B.1	North America / USA / Utah
20-023279-001_mink1_8-14-20_UT_F3_Index	Mink	8/14/20	B.1	North America / USA / Utah
20-023279-002_mink2_8-14-20_UT_F3_Index	Mink	8/14/20	B.1	North America / USA / Utah
20-023279-003_mink1_8-14-20_UT_F4_Index	Mink	8/14/20	B.1	North America / USA / Utah
20-023279-004_mink2_8-14-20_UT_F4_Index	Mink	8/14/20	B.1	North America / USA / Utah
20-023894-003_mink1_8-21-20_UT_F5_Index	Mink	8/21/20	B.1	North America / USA / Utah
20-023894-006_mink2_8-21-20_UT_F5_Index	Mink	8/21/20	B.1	North America / USA / Utah
20-026330-002v_mink1_9-14-20_UT_F6_Index	Mink	9/14/20	B.1	North America / USA / Utah
20-026330-003_mink2_9-14-20_UT_F6_Index	Mink	9/14/20	B.1	North America / USA / Utah
20-026330-003v_mink2_9-14-20_UT_F6_Index	Mink	unknown	unknown	unknown
20-026330-006_mink3_9-14-20_UT_F6_Index	Mink	unknown	unknown	unknown
20-027447-002_mink1_9-22-20_UT_F8_Index	Mink	9/22/20	B.1	North America / USA / Utah
20-027447-004_mink2_9-22-20_UT_F8_Index	Mink	9/22/20	B.1	North America / USA / Utah
20-027448-002_mink1_9-22-20_UT_F7_Index	Mink	9/22/20	B.1	North America / USA / Utah
20-027448-002v_mink1_9-22-20_UT_F7_Index	Mink	unknown	unknown	unknown
20-027448-004_mink2_9-22-20_UT_F7_Index	Mink	9/22/20	B.1	North America / USA / Utah
20-028629-002_minkA_9-28-20_MI_F1_Index	Mink	9/28/20	B.1	North America / USA / Michigan
20-028629-004_minkB_9-28-20_MI_F1_Index	Mink	9/28/20	B.1	North America / USA / Michigan
20-028674-001_mink1_10-1-20_UT_F9_Index	Mink	10/1/20	B.1	North America / USA / Utah
20-028674-001v_mink1_10-1-20_UT_F9_Index	Mink	unknown	unknown	unknown
20-028674-005_mink2_10-1-20_UT_F9_Index	Mink	10/1/20	B.1	North America / USA / Utah
20-028748-001_mink1_10-2-20_WI_F1_Index	Mink	10/2/20	B.1	North America / USA / Wisconsin
20-028748-002_mink2_10-2-20_WI_F1_Index	Mink	10/2/20	B.1	North America / USA / Wisconsin
20-028748-002v_mink2_10-2-20_WI_F1_Index	Mink	unknown	unknown	unknown
20-028748-006v_mink6_10-2-20_WI_F1_Index	Mink	unknown	unknown	unknown

Tree node ID	Host	Collection date	Lineage	Location
20-031493-001_mink1_10-29-20_WI_F2_Index	Mink	10/28/20	B.1	North America / USA / Wisconsin
20-031493-003_mink3_10-29-20_WI_F2_Index	Mink	10/28/20	B.1	North America / USA / Wisconsin
20-031893-001_mink_10-29-20_UT_F11_Index	Mink	10/29/20	B.1	North America / USA / Utah
20-031893-010_mink_10-29-20_UT_F11_Index	Mink	10/29/20	B.1	North America / USA / Utah
20-031894-014_mink_10-29-20_UT_F10_Index	Mink	10/29/20	B.1	North America / USA / Utah
20-031894-017_mink_10-29-20_UT_F10_Index	Mink	10/29/20	B.1	North America / USA / Utah
20-032048-005_mink_10-29-20_UT_F12b_Index	Mink	unknown	unknown	unknown
20-032048-008_mink_10-29-20_UT_F12b_Index	Mink	10/29/20	B.1	North America / USA / Utah
20-032049-004_mink_10-29-20_UT_F12a_Index	Mink	10/29/20	B.1	North America / USA / Utah
20-032049-008_mink_10-29-20_UT_F12a_Index	Mink	10/29/20	B.1	North America / USA / Utah
hCoV-19-mouse-USA-IA-N501Y-MA30/2021-EPI_zc	Mouse	unknown	unknown	unknown
21-011485-003_zc	Otter	unknown	B.1.1.7	unknown
21-011485-003s_otter_04-15-21_GA_np	Otter	unknown	B.1.1.7	unknown
21-011485-005_zc	Otter	unknown	B.1.1.7	unknown
21-011485-005s_otter_04-15-21_GA_np	Otter	unknown	B.1.1.7	unknown
21-011485-007s_otter_04-15-21_GA_np	Otter	unknown	B.1.1.7	unknown
21-011485-007v_otter_04-15-21_GA_np	Otter	unknown	B.1.1.7	unknown
21-011485-009s_otter_04-15-21_GA_np	Otter	unknown	B.1.1.7	unknown
21-011485-009v_otter_04-15-21_GA_np	Otter	unknown	B.1.1.7	unknown
21-011485-011s_otter_04-15-21_GA_np	Otter	unknown	B.1.1.7	unknown
21-011485-011v_otter_04-15-21_GA_np	Otter	unknown	B.1.1.7	unknown
20-035685-003_Snowleopard_12-4-20_KY_fc	Snow leopard	unknown	B.1.2	unknown
20-031353-001_tiger_10-9-20_TN	Tiger	10/19/20	B.1.2	North America / USA / Tennessee
20-031353-002_tiger_10-9-20_TN	Tiger	10/19/20	B.1.2	North America / USA / Tennessee
20-031498-003_tiger_10-27-20_TN_zoo	Tiger	10/27/20	B.1.2	North America / USA / Tennessee
21-001483-001s_tiger_01-10-21_MN_na	Tiger	1/10/21	B.1.564	North America / USA / Minnesota
21-002026-001s_tiger_01-16-21_TX_fc	Tiger	1/16/21	B.1.234	North America / USA / Texas
21-003442-002v_tiger_02-02-21_IN_fc	Tiger	2/2/21	B.1.2	North America / USA / Indiana
21-010728-001s_zc	Tiger	4/9/21	B.1.1.7	North America / USA / Virginia
21-010728-003s_tiger_04-09-21_VA_np	Tiger	unknown	unknown	unknown
21-010728-003v_tiger_04-09-21_VA_np	Tiger	unknown	unknown	unknown
root	Human		Root	China / Wuhan / NC_045512
